# Healthier rhythm, healthier brain? Integrity of circadian melatonin and temperature rhythms relates to the clinical state of brain‐injured patients

**DOI:** 10.1111/ene.13935

**Published:** 2019-03-19

**Authors:** C. Blume, M. Angerer, M. Raml, R. del Giudice, N. Santhi, G. Pichler, A. B. Kunz, M. Scarpatetti, E. Trinka, M. Schabus

**Affiliations:** ^1^ Department of Psychology University of Salzburg Laboratory for Sleep, Cognition, and Consciousness Research Salzburg Austria; ^2^ University of Salzburg Centre for Cognitive Neuroscience Salzburg Austria; ^3^ Surrey Sleep Research Centre Faculty of Health and Medical Sciences University of Surrey Guildford UK; ^4^ Geriatric Health Centres of the City of Graz Albert Schweitzer Clinic Apallic Care Unit Graz Austria; ^5^ Department of Neurology Paracelsus Medical University Christian Doppler Medical Center Salzburg Austria; ^6^ Gunther Ladurner Nursing Home Salzburg Austria

**Keywords:** brain injury, circadian rhythms, disorders of consciousness, neuropsychological assessment

## Abstract

**Background:**

Healthy circadian rhythmicity has been suggested to relate to a better state of brain‐injured patients and to support the emergence of consciousness in patient groups characterized by a relative instability thereof such as patients with disorders of consciousness (DOC).

**Methods:**

Going beyond earlier studies, a systems‐level perspective was adopted and, using multilevel modelling, the joint predictive value of three indices of circadian rhythm integrity derived from skin temperature variations, melatoninsulfate secretion, and physical activity (wrist actigraphy) patterns was evaluated for the behaviourally assessed state [Coma Recovery Scale ‐ Revised (CRS‐R) score] of DOC patients [13 unresponsive wakefulness syndrome; seven minimally conscious (exit) state]. Additionally, it was assessed in a subset of 16 patients whether patients’ behavioural repertoire (CRS‐R score) varied (i) with time of day or (ii) offset from the body temperature maximum (BT_max_), i.e. when cognitive performance is expected to peak.

**Results:**

The results reveal that better integrity of circadian melatoninsulfate and temperature rhythms relate to a richer behavioural repertoire. Moreover, higher CRS‐R scores are, by trend, related to assessments taking place at a later daytime or deviating less from the pre‐specified time of occurrence of BT_max_.

**Conclusions:**

In conclusion, the results suggest that therapeutic approaches aimed at improving circadian rhythms in brain‐injured patients are promising and should be implemented in hospitals or nursing homes. Beyond this, it might be helpful to schedule diagnostic procedures and therapies around the (pre‐assessed) BT_max_ (≈4 pm in healthy individuals) as this is when patients should be most responsive.

## Theoretical background

Variations in many biological and psychological processes follow a circadian pattern, i.e. they vary rhythmically with a period length of approximately 24 h. In healthy individuals, the existence of circadian variations in cognitive functions (e.g. 1; for a review see 2) and their underlying brain processes (e.g. 3) is well established. More recently, researchers have become interested in the relationship between circadian rhythms and clinical conditions when patients are in environments such as intensive care units or long‐term care homes, where night and day are often not clearly delineated 4, 5. Here, fairly low light levels during the day and relatively high light levels during the night, or both, may result in an impairment of sleep with detrimental effects on recovery from illness or immune responses (for a review see 6). Besides this, inappropriate entrainment may also bring about a relative instability of the sleep–wake cycle with frequent transitions between sleep/unconsciousness and wakefulness/consciousness occurring throughout the circadian (i.e. ≈24 h) cycle. This may be especially relevant in patients with disorders of consciousness (DOC), whose consciousness levels fluctuate strongly anyway. Amongst DOC, a distinction is made between the unresponsive wakefulness syndrome [UWS, formerly ‘vegetative state’ (VS); patients never show behavioural signs of consciousness despite phases of sleep and wakefulness] and the minimally conscious state (MCS; patients show inconsistent but reproducible signs of consciousness) 7. When patients regain the ability to functionally communicate/use objects, their state is denoted exit MCS (EMCS) 8. Consciousness is often described as comprising two components, wakefulness/arousal and awareness 9. Consolidated periods of wakefulness and sleep resulting from well‐entrained circadian rhythms seem crucial for adequate arousal levels and thus wakefulness, which is necessary for consciousness. Indeed, this hypothesis is supported by recent research from our group, where it was shown that the integrity of a patient's circadian rhythm is related to the arousal/wakefulness scores on the Coma Recovery Scale – Revised (CRS‐R) 10. Previously, research on the significance of circadian rhythms for consciousness in brain‐injured patients has looked at circadian variations in temperature 11, 12, cardiac parameters 13, 14 or hormones such as melatonin 15 and their relation to patients’ consciousness states. However, these studies have only considered single circadian parameters rather than looking at several manifestations of circadian rhythms in conjunction. From a systems‐level perspective, the interplay between different manifestations may therefore reveal a more comprehensive understanding.

Thus, the relationship between the patients’ state (CRS‐R scores) and circadian rhythm integrity indices of (i) body temperature, (ii) actigraphy‐derived activity patterns and (iii) melatonin secretion are investigated here using multilevel modelling. Aiming at a parsimonious model, one index was selected for each measure, which has been found to describe rhythm integrity well in previous research (cf. Supporting methods and materials for more details).

Beyond the analysis of the joint predictive value of three different circadian indices, variations in CRS‐R results across the day were also investigated. Previous research has suggested that the diagnosis may vary with daytime 16. Beyond this, it was speculated earlier that the temperature maximum might represent an ideal time point for CRS‐R assessments as cognitive performance is expected to peak at this time, which in the average healthy person occurs at about 4 pm (for a review see 2, 17). A second aim was therefore to investigate if and how the patients’ state (as assessed with the CRS‐R) varies as a function of daytime and/or time offset from the temperature maximum.

## Methods and materials

### Patients

A total of 23 patients from long‐term care facilities were included in the study. Measurements of urinary 6‐sulfatoxymelatonin (aMT6s) levels were obtained in 20 patients and multiple examinations with the CRS‐R in 16. Informed consent was obtained from the patients’ legal representatives, and the study was approved by the local ethics committees of the Land Salzburg and the Medical University Graz. Table 1gives more details on the study sample.

**Table 1:** Demographic information and circadian parameters

Patient ID	Multiple CRS‐R assessments	Skin temperature (period length)	Actigraphy (interdaily stability)	Melatonin (fit of the BCF, *R* ^2^)	Time since injury (months)	Age at incident	Gender	Aetiology
P1	✓	24.39	0.2	81.82	10	71.2	F	NTBI
P2	✓	23.79	0.15	76.54	15	32.75	M	TBI
P3	✓	24.52	0.21	83.80	16	47.7	F	NTBI
P4	✓	24.34	0.19	48.53	6	58.5	F	NTBI
P5	✓	24.29	0.23	81.82	5	67.6	M	NTBI
P6	–	–	–	68.39	13	68.9	F	TBI
P7	✓	24.22	0.14	74.73	37	44.9	F	NTBI
P8	✓	23.48	0.15	93.70	56	15.3	M	TBI
P9	✓	24.26	0.21	73.68	168	41	F	TBI
P10	–	23.60	0.18	57.50	15	68.75	F	NTBI
P11	✓	23.64	0.37	19.25^a^ (n.s.)	54	46.5	M	TBI
P12	–	23.64	0.13	87.68	415	33.4	M	NTBI
P13	–	23.60	0.37	82.30	10.5	52.1	F	NTBI
P14	–	24.90	0.23	28.40^a^ (n.s.)	13.5	66.9	F	TBI
P15	–	24.39	0.17	75.24	2.5	70.8	F	TBI
P16	✓	24.87	0.15	77.90	82	46.2	F	NTBI
P17	✓	23.69	0.20	63.36	197	20.6	M	TBI
P18	–	23.26	–	59.66	2	67.8	M	TBI
P19	✓	24.06	0.16	78.70	3	45.75	F	NTBI
P20	✓	23.96	0.18	42.50	13	76.9	M	NTBI
P21	✓	23.38	0.33	–	17	17.6	F	TBI
P22	✓	19.86	0.08	–	1.5	76.9	M	NTBI
P23	✓	23.62	0.13	–	10	26.9	M	TBI

BCF, baseline cosine function; CRS‐R, Coma Recovery Scale – Revised. Period length is the length of the circadian skin temperature cycle. Interdaily stability (IS) is a score informing about how well the patients’ rest–activity cycles were entrained to the light–dark cycle (range 0–1 with 1 reflecting perfect IS). Multiple CRS‐R assessments were not available for all patients as they were only added to the protocol later. In some patients, urine could not be sampled to obtain melatoninsulfate levels as they did not have a catheter. Missing values for skin temperature or actigraphy are due to technical failure. ^a^The BCF fit was not significant in this patient.

### Experimental design

**Figure 1:**
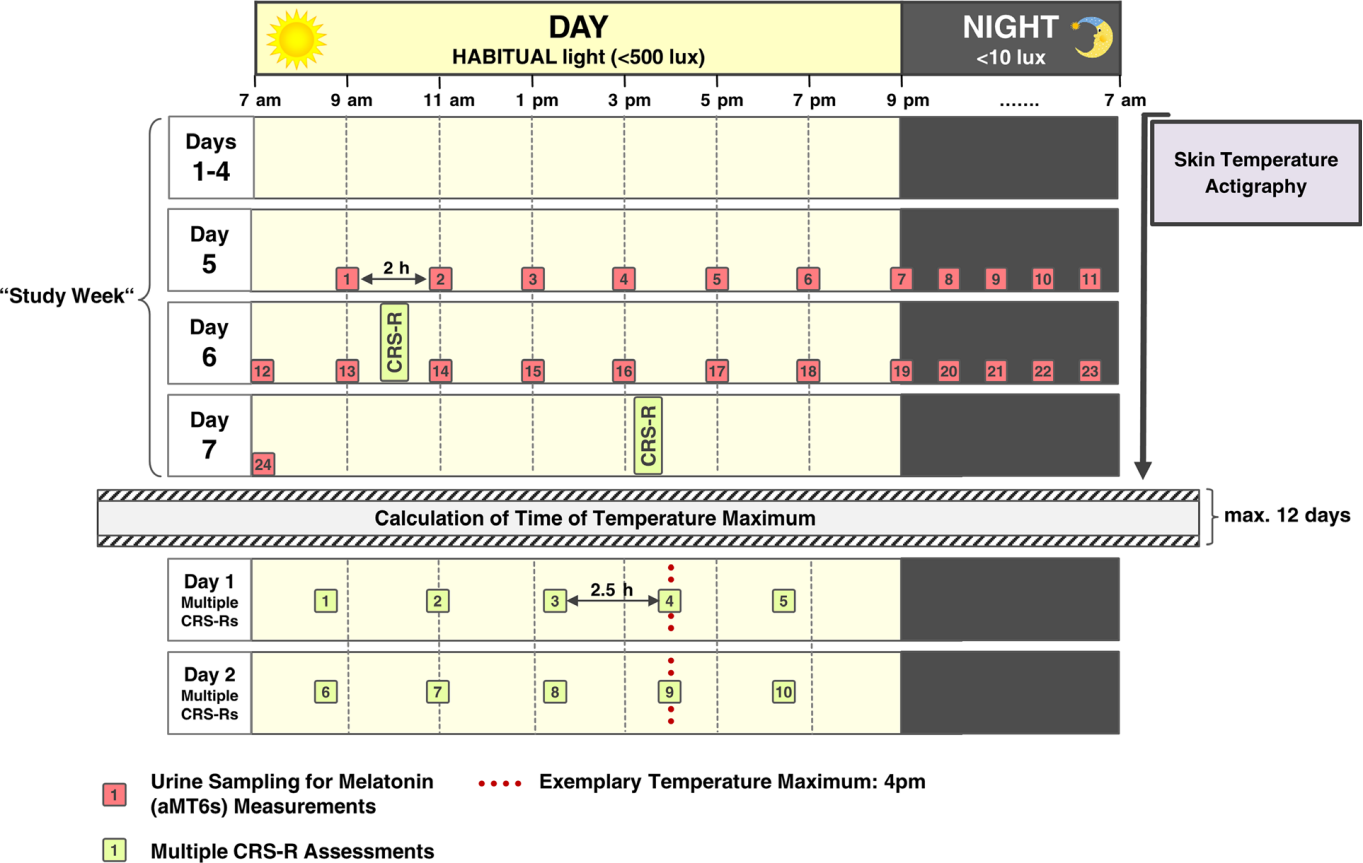
Overview of the study protocol. Skin temperature variations and actigraphy were recorded over the course of seven days and urinary 6‐sulfatoxymelatonin (aMT6s) levels were measured in 2‐h intervals during 48 h on days 5/6 of the study week. Ambient light levels were recorded with the actigraphs and confirmed by spot checks at eye level. Following the study week, the time of occurrence of the temperature maximum was calculated and multiple CRS‐R assessments were scheduled according to this time. Patients were then repeatedly assessed at the time of the temperature maximum as well as at multiples of 2.5 h before and after this time point between 7 am and 9 pm on two consecutive days. [Colour figure can be viewed at wileyonlinelibrary.com]

The study protocol comprised seven full days (called the ‘study week’ henceforth) during which actigraphy and skin temperature were measured continuously. Urine samples for aMT6s measurements were taken every 2 h on days 5 and 6 of the study week. At the end of this week, patients’ behavioural repertoire/state was assessed in two behavioural examinations with the CRS‐R 10. Following this week, the time of occurrence of the body temperature maximum was calculated. Patients were then again assessed on two consecutive days repeatedly with the CRS‐R (time points regularly spaced around the temperature maximum). During the whole protocol, patients stayed in their habitual hospital environment. Figure 1 gives an overview of the protocol, and the Supporting methods and materials give more details.

### Behavioural assessment

Patients’ neuropsychological state was assessed with the CRS‐R and the score deemed ‘most representative’ of his/her state was selected. For more details see Supporting methods and materials. Tables 2 and S1 provide an overview of the CRS‐R assessment results during the study week, and Tables 2 and S3 for the assessments according to the (pre‐determined) temperature maximum.

**Table 2:** Results of the CRS‐R assessments during the study week and for subsequent multiple assessments according to the temperature maximum

Patient ID	Aetiology	Study week diagnosis	Study week CRS‐R sum score	Temperature maximum time	Multiple assessment diagnoses	Multiple assessment CRS‐R sum score
P1	NTBI	VS/UWS	6	16:50	VS/VS/VS/**VS**/VS	5/5/5/**5**/6
P2	TBI	VS/UWS	5	16:20	VS/VS/VS/**VS**/VS	4/2/6/**4**/3
P3	NTBI	VS/UWS	5	13:40	VS/**VS**/VS/VS/VS	6/**5**/5/4/5
P4	NTBI	VS/UWS	4	12:10	VS/**VS**/VS/VS/VS	5/**5**/5/6/6
P5	NTBI	VS/UWS	6	14:50	VS/VS/**VS**/VS/VS	5/4/**3**/3/2
P6	TBI	VS/UWS	2	–	–	–
P7	NTBI	VS/UWS	5	06:05	VS/VS/VS/VS/VS	3/4/3/2/3
P8	TBI	MCS	13	21:15	VS/VS/MCS/VS/MCS	4/4/7/5/9
P9	TBI	MCS	17	16:00	MCS/MCS/MCS/**MCS**/MCS	8/8/12/**9**/11
P10	NTBI	VS/UWS	3	–	–	–
P11	TBI	VS/UWS	4	12:10	VS/**VS**/VS/VS/VS	4/**4**/4/3/4
P12	NTBI	VS/UWS	4	–	–	–
P13	NTBI	MCS	13	–	–	–
P14	TBI	MCS	9	–	–	–
P15	TBI	EMCS	23	–	–	–
P16	NTBI	VS/UWS	5	16:30	VS/VS/VS/**VS**/VS	3/3/4/**4**/4
P17	TBI	MCS	9	17:50	MCS/MCS/MCS/MCS/**MCS**	8/8/8/8/**8**
P18	TBI	VS/UWS	2	–	–	–
P19	NTBI	VS/UWS	3	13:50	VS/VS/**VS**/VS/VS	4/3/**4**/3/4
P20	NTBI	MCS	7	11:55	VS/**MCS**/MCS/MCS/MCS	3/**9**/7/7/7
P21	TBI	VS/UWS	5	10:15	VS/**MCS**/VS/MCS/MCS	4/**8**/4/6/8
P22	NTBI	VS/UWS	2	18:15	VS/VS/VS/VS/**VS**	3/2/2/4/**2**
P23	TBI	EMCS	20	14:00	EMCS/EMCS/**EMCS**/EMCS/EMCS	20/20/**20**/20/20

The diagnoses and sum scores obtained during assessments at the time of the temperature maximum are marked in bold. The assessment results to the left/right of the rating in bold represent further assessments that were scheduled at intervals of 2.5 h around the time of the maximum. Note that for P7 and P8 the temperature maximum occurred at times when an assessment was not possible. For P7 and P8 the assessments closest to the temperature maximum are therefore the first and the last ones, respectively. CRS‐R, Coma Recovery Scale – Revised; EMCS, Exit MCS; MCS, minimally conscious state; NTBI, non‐traumatic brain injury; TBI, traumatic brain injury; VS/UWS, vegetative state/unresponsive wakefulness syndrome.

For actigraphy, following careful pre‐processing the interdaily stability (IS) (cf. Tables 1 and S2), which reflects how well the patients’ activity rhythms were entrained to a 24 h zeitgeber, was calculated. The Supporting methods and materials give more details.

### Physiological assessments

Skin temperature was sampled using four external skin sensors. To find the length of each patient's circadian skin temperature rhythm, Lomb–Scargle periodograms 18, 19 were computed using a proximal–distal skin temperature gradient (for details see the Supporting methods and materials).

For the multiple CRS‐R assessments according to the pre‐assessed temperature maximum its time of occurrence was calculated and five assessments on each of two consecutive days in 2.5 h intervals around the peak were scheduled (cf. the section ‘Behavioural assessment’ and Fig. 1). For more details see the Supporting methods and materials.

**Figure 2:**
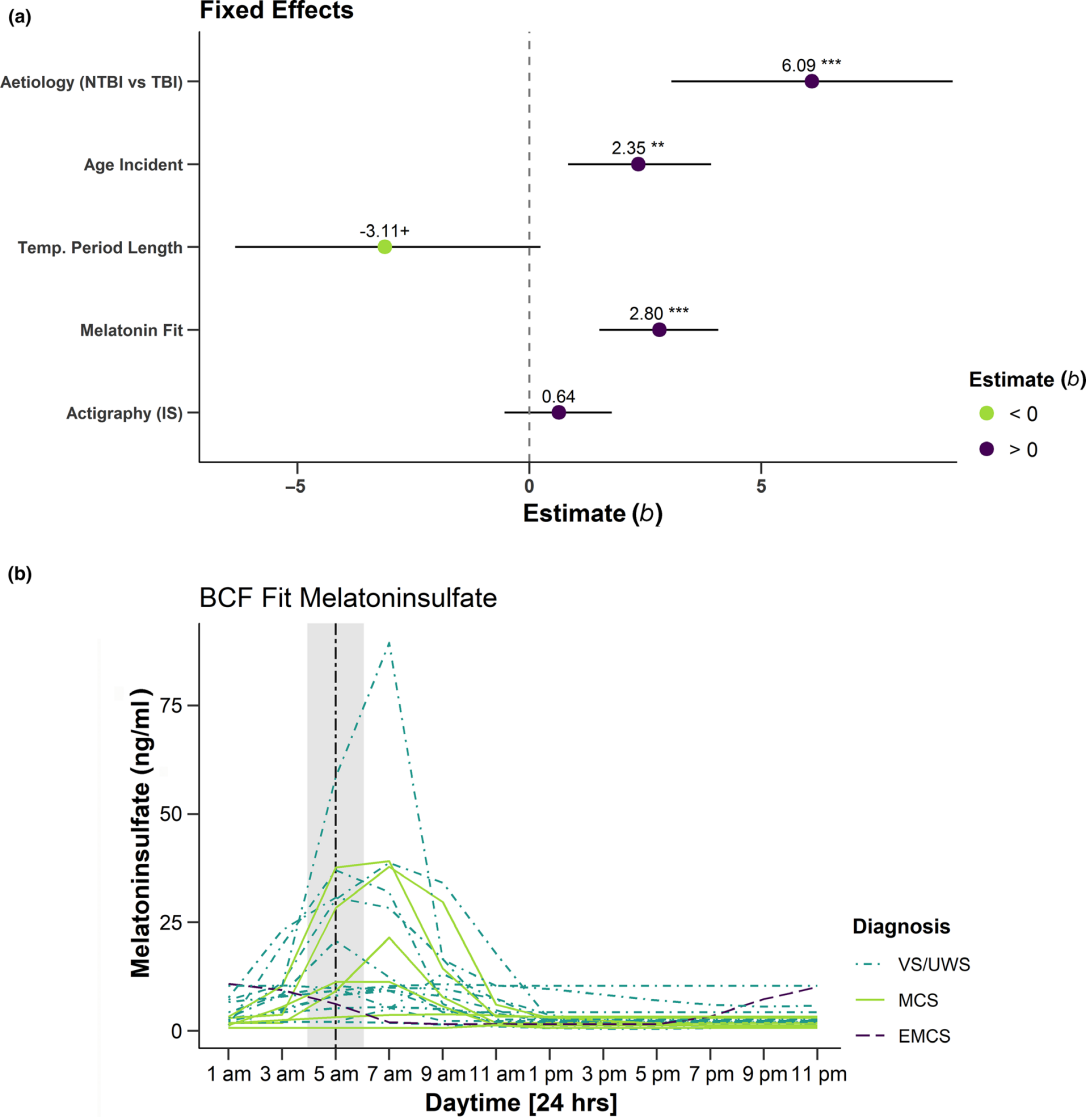
(a) Predictions [estimates (*b*)] for fixed effects [i.e., aetiology, age at incident, deviation of the period length from 24.18 h, fit of the BCF function, interdaily stability (IS) of actigraphy values] on CRS‐R sum scores (Model 1). The figure illustrates that TBI, a higher age at the incident, a ‘healthier’ melatonin secretion as indicated by a better fit to the BCF function and, by trend, less deviation of the period length of the skin temperature variations from a ‘healthy’ standard (24.18 h) predicted a higher CRS‐R sum score. Horizontal bars represent 95% confidence intervals. Asterisks indicate significance, +*p* < 0.01, *p < 0.05, **p < 0.01, ***p < 0.001. (b) Results of the fit of the BCF function to the melatoninsulfate data. For all participants the secretion peaks in the (early) morning hours. The grey shaded area indicates the time during which melatoninsulfate is expected to peak in healthy individuals, i.e. between 4 and 6am with an estimated delay of about two hours relative to the peak of plasma melatonin levels. *b*, standardised regression coefficient; BCF, Baseline Cosine Function; (N)TBI, (non‐)traumatic brain injury. [Colour figure can be viewed at wileyonlinelibrary.com]

For the circadian melatonin rhythm, variations in aMT6s levels in urine were analysed and a baseline cosine function (BCF) was fitted to the data. For a detailed overview of the BCF fit results and associated parameters see Table S1 and Fig. 2b; see the Supporting information for methodological details.

### Statistical analyses

For all analyses, R version 3.4.3 20 was used. Discrete variables were *z*‐transformed. Effects with *P* values <0.05 are denoted significant, effects with 0.05 < *P* < 0.1 trends. For details see the Supporting methods and materials.

#### Model 1

Model 1 assessed the relationship between the patients’ behavioural state [(best) CRS‐R sum score during the study week] and indices of circadian rhythm integrity of (i) skin temperature, (ii) actigraphy and (iii) aMT6s secretion (fixed effects). The diagnosis (i.e. VS/UWS, MCS or EMCS) was modelled as a random intercept. For (i) the absolute deviation of the period length from 24.18 h, i.e. the average period length in healthy individuals 21, was included. For (ii) the IS indicating how well the rest–activity rhythm was entrained to the light–dark cycle was included 22. Lastly, for (iii) the Fisher *z*‐transformed *R* ^2^ of the BCF fit was included. In addition, the patients’ age at the time of the injury and aetiology [i.e. traumatic brain injury (TBI) versus non‐traumatic brain injury (NTBI)], which have previously been shown to relate to diagnosis and prognosis, were also included as fixed effects.

#### Models 2a, 2b

A second model assessed whether the patients’ behavioural state varied as a function of time offset from the temperature maximum and the time of day; these have previously been related to the patients’ state 11, 16. To this end, the sum scores of repeated CRS‐R assessments were modelled. As in model 1, aetiology and age at incident were included as fixed factors. Daytime was included as a linear trend (model 2a). In model 2b, daytime was replaced by the time offset from the temperature maximum, which was included as a quadratic trend.

## Results

Analyses of differences between the diagnostic subgroups (i.e. VS/UWS and MCS/EMCS) or the aetiology subgroups (i.e. TBI and NTBI) in circadian indices of temperature, melatonin and actigraphy did not indicate any differences. For details see the Supporting results.

### Model 1

Coma Recovery Scale – Revised sum scores varied in intercepts across diagnoses (i.e. VS/UWS versus MCS/EMCS) with an MCS/EMCS diagnosis being associated with higher scores, SD = 2.92 [95% confidence interval (CI) 0.6, 9.55], *χ* ^2^(1) = 9.76, *P* = 0.002. Moreover, aetiology (*b* = 6.09, *t*(17) = 4.17, *P* < 0.001) and age at incident (*b* *=* 2.35, *t*(16) = 2.68, *P* = 0.007, SD_age_ = 19.6 years) predicted CRS‐R sum scores (NTBI was associated with lower CRS‐R scores than TBI). Besides, the fit of the BCF to aMT6s levels (for fitted curves see Fig. 2b) contributed to predicting CRS‐R scores (*b* = 2.80, *t*(16) = 4.37, *P* < 0.001, SD_R2(aMT6s)_ = 0.38). The deviation of the period length of the patients’ temperature rhythms from 24.18 h contributed with a trend (*b* = −3.11, *t*(16) = −1.87, *P* = 0.081, SD_deviation_ = 0.72). The actigraphy‐derived IS did not predict the CRS‐R sum score (*t*(16) = 1.07, *P* = 0.3). For an illustration of the effects see Fig. 2a. Table S5 provides an overview of the results and the contribution of each factor to the model in terms of explained variance (*R* ^2^).

### Models 2

**Figure 3:**
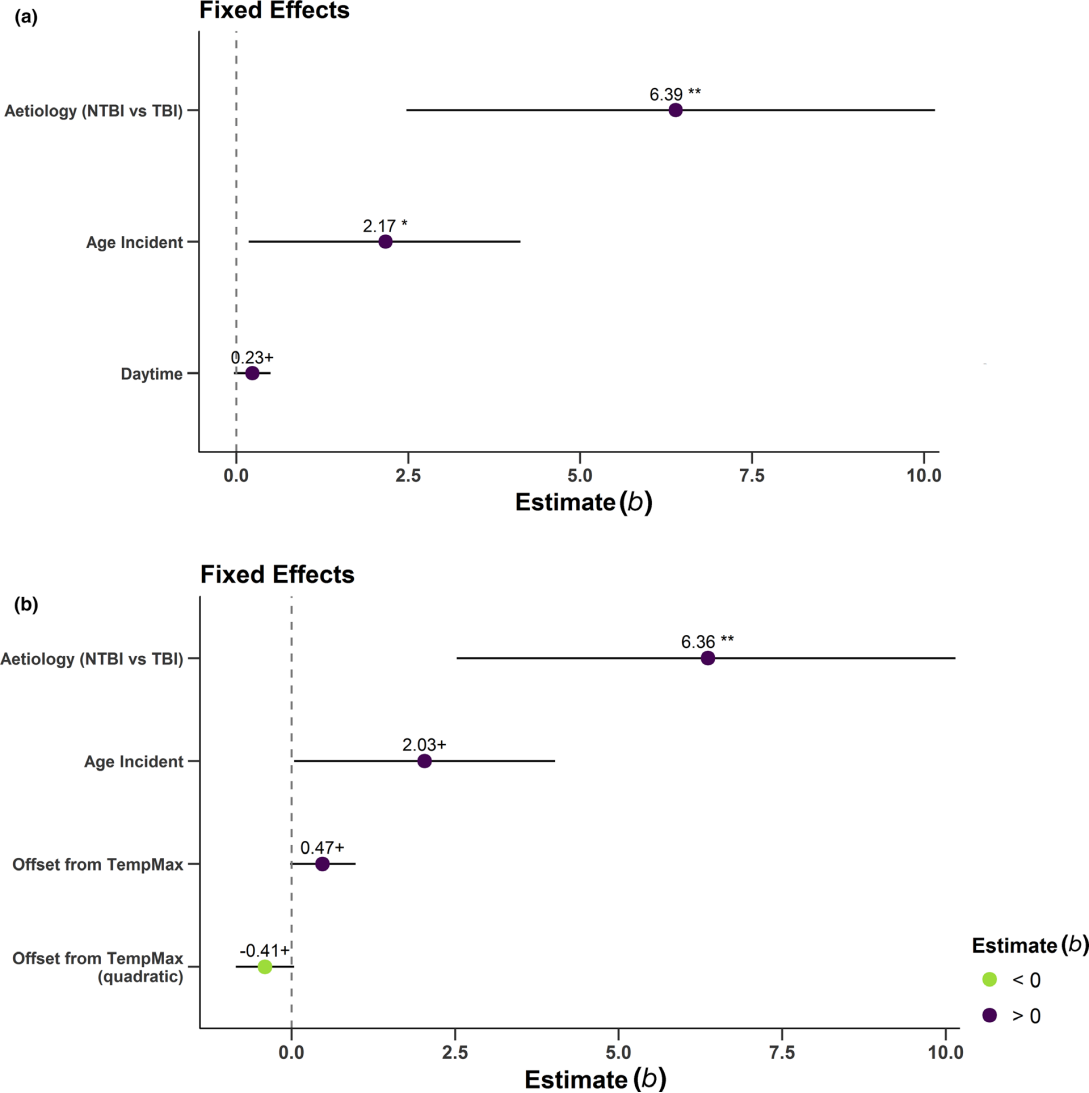
Predictions for fixed effects in Models 2a, 2b. (a) Predictions [estimates (*b*)] for fixed effects of aetiology, age at incident and daytime on CRS‐R sum scores (Model 2a). The figure illustrates that TBI, a higher age at the time of incident and, by trend, a later daytime, predict higher CRS‐R sum scores. (b) Predictions [estimates (*b*)] for fixed effects of aetiology, age at incident and offset from the temperature maximum as a quadratic function on CRS‐R sum scores (Model 2b). The figure illustrates that TBI and, by trend, higher age at incident and less deviation from the temperature maximum, predict higher CRS‐R scores. Horizontal bars represent 95% confidence intervals. Asterisks indicate significance: +*P* < 0.1, *P < 0.05, **P < 0.01. *b*, standardized regression coefficient; NTBI, non‐traumatic brain injury; TBI, traumatic brain injury. [Colour figure can be viewed at wileyonlinelibrary.com]

In model 2a, intercepts varied across participants with an SD of 3.11 (95% CI 2.25, 4.64). Moreover, again aetiology (*b* = 6.39, *t*(16) = 3.24, *P* = 0.005) and age at incident (*b* = 2.17, *t*(16) = 2.15, *P* = 0.048, SD_age_ = 18.1 years) predicted CRS‐R sum scores. The daytime also predicted CRS‐R scores; however, this was only significant by trend (*b* = 0.23, *t*(64) = 1.73, *P* = 0.088, SD_time of day_ = 3.76). Figure 3a and Table S6 give an overview of the effects in model 2a.

For model 2b, intercepts also varied across participants [SD = 3.13 (95% CI 2.26, 4.66)] and aetiology predicted CRS‐R scores (*b* = 6.36, *t*(16) = 3.21, *P* = 0.006). As for all models, NTBI aetiology was associated with lower CRS‐R scores than TBI. Age at incident (*b* = 2.03, *t*(16) = 2.00, *P* = 0.063, SD_age_ = 18.1 years) was only significant by trend. The quadratic effect of the time offset from the temperature maximum was also significant by trend (*b* = −0.41, *t*(65) = −1.77, *P* = 0.082) and indicated an inverted U‐shaped relationship, i.e. less deviation from the temperature maximum was associated with higher CRS‐R scores. Figure 3b and Table S7 give an overview of the effects in model 2b. A comparison of models 2a and 2b did not indicate a difference, *χ* ^2^(1) = 1.57, *P* = 0.21. Results for a third model (2c) including both time offset from the temperature maximum and time of day can be found in the supporting results (Table S8).

## Discussion

Adopting a systems‐level perspective, this study provides novel evidence for the significance of intact circadian rhythmicity for the state of severely brain‐injured patients. Moreover, the data suggest that the result of assessments of the patients’ state may depend on when they take place; therefore it may be helpful to have their timing guided by a circadian index such as patients’ body temperature or by the time of day.

Whilst earlier studies focused on isolated circadian parameters and their relationship with the patients’ state, using multilevel modelling, the joint predictive quality of three different indices of circadian rhythm integrity derived from (i) body temperature, (ii) actigraphy‐derived activity and (iii) melatonin secretion, was investigated here. Amongst these, higher integrity of the melatonin rhythm and less deviation of the temperature rhythm from the supposedly ‘healthy’ period length of 24.18 h were related to higher CRS‐R scores. In line with earlier findings 11, 15, this first of all provides further support for the notion of the significance of circadian rhythms in severely brain‐injured patients. More precisely, besides awareness, adequate arousal levels are seen as a necessary background condition for consciousness 9. Disturbances of circadian rhythmicity that affect the stability of the sleep–wake cycle may result in frequent transitions between wakefulness and sleep. The resulting relative instability of arousal and thus consciousness levels should be especially critical in patients with DOC whose consciousness levels fluctuate strongly anyway. In contrast to melatonin and body temperature and also contrasting earlier findings 23, circadian variations in physical activity were not related to CRS‐R scores in the model. However, the study by Cruse and colleagues 23 solely assessed the existence of truly circadian (i.e. ≈24 h) activity patterns, which may, at least partly, account for the deviating findings. Moreover, the authors did not correct for movement artefacts by for example nursing. Besides this, the usefulness of actigraphy in brain‐injured patients may be hampered by motor activity often being impaired, e.g. due to spasticity or the use of muscle relaxants. Beyond circadian indices, also age at onset and the aetiology were significantly related to the patients’ state. Specifically, traumatic aetiology and higher age at the time of injury predicted a higher CRS‐R score in the model. Whilst the finding regarding aetiology is well in line with common expectations, one would rather expect younger age to be related to higher scores (aetiology 24, 25, age 26). One explanation could be that the relationship between age and neuroplasticity seems to be moderated by aetiology 27. Moreover, the TBI and NTBI groups were both rather middle‐aged at the time of injury and did not differ (median_TBI_ = 46.5 years; median_NTBI_ = 52.1 years; ANOVA‐type statistic_1,18_ = 0.7, *P* = 0.41).

Beyond the three circadian indices, a second model assessed whether daytime and/or the time offset from the body temperature maximum could predict the patients’ behavioural state. Intriguingly, daytime and the offset from the temperature maximum were related to the patients’ state, although only by trend. This first suggests that the behavioural state seems to vary throughout the day. Specifically, results suggest that later daytime and less offset from the temperature maximum were associated with higher CRS‐R scores. Note that, although the effect was not overly strong, it may still be decisive for whether consciousness is attested or not. The findings are in line with speculations in an earlier study from our group 11 and suggest that cognitive performance peaks around the time of the temperature maximum, i.e. around 4 pm in healthy individuals, median of the study sample 2:25 pm. (for a review see 2, 17). Admittedly, though, results contrast earlier findings by Cortese *et al*. who had found an MCS diagnosis to be more likely in the morning 16.This may be explained by methodological differences such as the lack of control of ambient light levels. Unfortunately, the relative contributions of daytime and time offset from the temperature maximum are impossible to disentangle as they are naturally highly correlated.

A possible limitation of the present study is that it was not possible to include neuroimaging data to evaluate differences in brain injury and potential damage to the suprachiasmatic nuclei. Also, future studies will have to replicate the findings in larger samples possibly requiring multicentric studies and include more central indices of circadian rhythm integrity derived from for example electroencephalography.

To conclude, this study provides novel evidence from a systems‐level perspective for the relevance of circadian rhythm integrity for the state of brain‐injured patients. Thereby, it also makes a case for interventions that aim at normalizing circadian rhythmicity, which may include the vespertine administration of melatonin and/or the use of light to more clearly delineate night and day. Moreover, the results tentatively suggest that it may be helpful to schedule neuropsychological assessments around the time of the body temperature maximum, which would allow for an individualized pre‐specification of the optimal test time. If not viable, it is recommended that assessments preferably take place in the afternoon around 4 pm.

## Disclosure of conflicts of interest

The authors declare no financial or other conflicts of interest.

## Supporting information


**Figure S1.** Boxplot of the distribution of period lengths of circadian temperature rhythms according to the consciousness state.
**Figure S2.** Boxplot of the distribution of period lengths of circadian temperature rhythms according to the aetiology.
**Figure S3.** Boxplot of the distribution of the fit of the baseline cosine function according to the consciousness state.
**Figure S4.** Boxplot of the distribution of the fit of the baseline cosine function according to the aetiology.
**Figure S5.** Boxplot of the distribution of the interdaily stability (IS) of actigraphy patterns according to the diagnosis.
**Figure S6.** Boxplot of the distribution of the interdaily stability (IS) of actigraphy patterns according to the aetiology.
**Table S1.** Detailed results of the fit of the baseline cosine function (BCF) to the melatoninsulfate (aMT6s) data.
**Table S2.** Detailed results of the analysis of the actigraphy data.
**Table S3.** Detailed results of the CRS‐R assessments during the study week.
**Table S4.** Detailed results of the multiple CRS‐R assessments according to the temperature maximum.
**Table S5.** CRS‐R scores as predicted by aetiology (NTBI vs.TBI), age at incident, and the three circadian indices (Model 1).
**Table S6.** CRS‐R scores as predicted by aetiology (NTBI versus TBI), age at incident, and time of day (model 2a).
**Table S7.** CRS‐R scores as predicted by aetiology (NTBI vs. TBI), age at incident, and offset from the temperature maximum as a quadratic function (model 2b).
**Table S8.** CRS‐R scores as predicted by aetiology (NTBI versus TBI), age at incident, time of day, and offset from the temperature maximum as a quadratic function (model 2c).
