# Supplementary material for: Healthier rhythm, healthier brain? Integrity of circadian melatonin and temperature rhythms relates to the clinical state of brain‐injured patients
Source: Eur J Neurol. 2019 Mar 19;26(8):1051–9. doi: 10.1111/ene.13935 (PMC6766891; doi:10.1111/ene.13935)
Supplement: Supplementary file 1 — Figure S1. Boxplot of the distribution of period lengths of circadian temperature rhythms according to the consciousness state. Figure S2. Boxplot of the distribution of period lengths of circadian temperature rhythms according to the aetiology. Figure S3. Boxplot of the distribution of the fit of the baseline cosine function according to the consciousness state. Figure S4. Boxplot of the distribution of the fit of the baseline cosine function according to the aetiology. Figure S5. Boxplot of the distribution of the interdaily stability (IS) of actigraphy patterns according to the diagnosis. Figure S6. Boxplot of the distribution of the interdaily stability (IS) of actigraphy patterns according to the aetiology. Table S1. Detailed results of the fit of the baseline cosine function (BCF) to the melatoninsulfate (aMT6s) data. Table S2. Detailed results of the analysis of the actigraphy data. Table S3. Detailed results of the CRS‐R assessments during the study week. Table S4. Detailed results of the multiple CRS‐R assessments according to the temperature maximum. Table S5. CRS‐R scores as predicted by aetiology (NTBI vs.TBI), age at incident, and the three circadian indices (Model 1). Table S6. CRS‐R scores as predicted by aetiology (NTBI versus TBI), age at incident, and time of day (model 2a). Table S7. CRS‐R scores as predicted by aetiology (NTBI vs. TBI), age at incident, and offset from the temperature maximum as a quadratic function (model 2b). Table S8. CRS‐R scores as predicted by aetiology (NTBI versus TBI), age at incident, time of day, and offset from the temperature maximum as a quadratic function (model 2c). [file ENE-26-1051-s001.docx]

**Body Rhythms in Concert? Circadian Rhythm Integrity and Time of Day Predict the Clinical State in Brain-Injured Patients**

ONLINE ONLY - SUPPLEMENTARY MATERIAL

Dr Christine Blume^1,2^*, MSc Monika Angerer^1^, MSc Marion Raml^1^, Dr Renata del Giudice^1,2^, Dr Nayantara Santhi^3^, Gerald Pichler M.D.^4^, Alexander Kunz M.D. ^5,6^, Monika Scarpatetti M.D.^4^, Prof. Eugen Trinka M.D., MSc, FRCP^2,5^, Prof. Manuel Schabus^1,2^

^1^ University of Salzburg, Department of Psychology, Laboratory for Sleep, Cognition and Consciousness Research, Hellbrunner Strasse 34, 5020 Salzburg

^2^ University of Salzburg, Centre for Cognitive Neuroscience Salzburg (CCNS), Hellbrunner Strasse 34, 5020 Salzburg

^3^ Surrey Sleep Research Centre, Faculty of Health and Medical Sciences, University of Surrey, Egerton Road, Guildford GU2 7XP, United Kingdom

^4^ Geriatric Health Centres of the City of Graz, Albert Schweitzer Clinic, Apallic Care Unit, Albert-Schweitzer-Gasse 36, 8020 Graz, Austria

^5^ Department of Neurology, Christian Doppler Medical Center, Paracelsus Medical University Salzburg, Ignaz-Harrer-Strasse 79, 5020 Salzburg, Austria

^6^ Gunter Ladurner Nursing Home, Ignaz-Harrer-Str. 79, 5020 Salzburg, Austria

**Corresponding Author:**

Dr. Christine Blume

University of Salzburg

Centre for Cognitive Neuroscience (CCNS)

Laboratory for “Sleep, Cognition and Consciousness Research”

Hellbrunner Str. 34

A-5020 Salzburg

Email: [christine.blume@sbg.ac.at](mailto:christine.blume@sbg.ac.at)

T: +43(0)662 8044-5148

**Methods and Materials**

***Patients***

A total of *N* = 23 (13 women) patients aged 19-78 (*mdn* = 54 years) were included in the study sample. Fifteen patients were in VS/UWS, six in an MCS and two in an EMCS state. We deliberately also included EMCS patients to cover the full spectrum of DoC. Out of these 23, measurements of urinary 6-sulfatoxymelatonin (aMT6s) levels were obtained in 20 (12 women) participants and multiple examinations with the CRS-R were carried out in 16 patients (8 women). Table 1 provides an overview of what data were available from which patient. Out of the 23 patients the skin temperature data of three (P10, P13, P14) had been included in the study sample from Blume et al. (2017). We recruited patients from long-term care facilities in hospitals in Austria. At the time the measurements took place patients did not suffer from dysautonomia or acute illnesses. Moreover, they were free from medication such as melatonin that is known to affect circadian rhythms. All patients in the cooperating centres in Austria that fulfilled the criteria and for whom informed consent was obtained were included in the study. As magnetic resonance imaging (MRI) data were not accessible the extent of brain damage, which may have involved hypothalamic damage, is unclear. All patients presented with alternating periods of open and closed eyes, which could represent periods of wakefulness and sleep, respectively. Informed consent was obtained from the patients’ legal representatives and approval of the local ethics committee had been obtained. For some patients, patient’s legal representatives only conceded part of the experimental design, e.g. excluding melatonin measurements, explaining missing data points.

***Experimental design***

The study protocol comprised one week during which actigraphy, and skin temperature were assessed continuously. Urine samples for 6-sulfatoxymelatonin (aMT6s) measurement samples were taken every two hours between 7am on day 5 and 7am on day 7. Behavioural assessments with the Coma-Recovery Scale Revised (Kalmar & Giacino, 2005) took place on day 6 in the morning and day 7 in the afternoon. Following this week we calculated the time of occurrence of the body temperature maximum (for details see below) and repeatedly assessed patients on two days with the assessments taking place at different time intervals from the temperature maximum (please see Figure 1 for an overview of the study protocol).

During the whole protocol patients continued to be in their habitual hospital environment (including therapies, visits, nursing etc.). Only during the two days with multiple CRS-R measurements according to the temperature maximum patients were not mobilised and therapies were discontinued to not affect the measurements. During the study week the light in the patients’ rooms was switched on at 7am and off at 9pm to keep the light-dark cycle constant. This rhythm was easiest to implement in the hospital environment and closest to the usual light-dark cycle there although it slightly deviates from the normal 2:1 ratio of wakefulness and sleep that is usually present in healthy individuals (Cohen et al., 2010). Nurses moved the patients every 3-4 hours during day and night. During the day, i.e. between 7am and 9pm light levels were kept below 500 lux at eye level, which was confirmed by spot checks with a digital luxmeter (Dr. Meter, Digital Illuminance/Light Meter LX1330B). Moreover, light levels were tracked by continuous recordings of ambient light levels with actigraphs (wGT3X-BT Monitor, ActiGraph LLC., Pensacola, USA) that were placed at the bedhead in a way their orientation matched the patients’ perspective as closely as possible. Please note though that the actigraphs are characterised by reduced sensitivity especially at low light levels and it cannot be excluded that they were for example inadvertently and unnoticedly covered by bed sheets between regular checks by the research team. Patients were not exposed to sunlight or wore dark sunglasses covering the whole eye when having to leave the building e.g. for medical reasons.

During the nights, it was necessary to switch on a dim light (< 10 lux at patients’ eye level) when nurses entered the patient’s room (usually twice per night for approx. 5-10 minutes) with patients being in complete darkness for the rest of the time. To ensure low light levels even when the sun rose earlier than 7am during the summer months blinds were closed during the lights off period. Patients received nutrition on a more or less constant level with breaks being e.g. due nurses having to replace empty nutrition bags via a stomach tube.

***Behavioural Assessment and Data Analysis***

*Coma-Recovery-Scale Revised*

Patients were assessed behaviourally with the Coma-Recovery-Scale Revised (CRS-R, Kalmar & Giacino, 2005) by two trained experts in the morning of day six and in the afternoon of day seven of the study week. Besides this, they were assessed repeatedly on two days that were separated from the study week by a maximum of seven days. On these two days, they were assessed in regular time intervals spaced by 2.5h around the time of occurrence of the temperature maximum, which had previously been calculated from the data from the “study week”. The CRS-R is widely acknowledged as the best available tool for the neuropsychological examination of DOC patients. It comprises 23 items that are grouped into six subscales reflecting auditory, visual, motor, oromotor, communication and arousal functions. Items of each subscale form a hierarchy with the lowest item representing only reflexive activity that indicates brain stem functioning. The highest item represents cognitively mediated behaviours involving cortical processes. Patients are tested in a hierarchical manner starting with the highest item of each subscale and the examiner then moves down the scale until the patient’s response meets the criteria for one item. This way a patient receives a specific score on each subscale. The scores of all subscales sum up to a maximum score of 23. For our analyses of the “study week” data, we compared the scores obtained in the two assessments of the “study week” and selected the assessment with the best diagnosis or highest sum score as this is thought to best represent the true state of the patient. The same selection method was used to select one assessment result for each time of day from the multiple CRS-R assessments according to the temperature maximum, which resulted in a selection of five assessments. For an overview of the CRS-R assessment results during the study week please see Table 2 and Suppl. Table 1 for detailed results. For the results of the multiple assessments according to the temperature maximum please also see Table 2 as well as Suppl. Table 2.

*Actigraphy*

We recorded actigraphy during the “study week” using the GT3X+ devices (ActiGraph LLC., Pensacola, FL 32502). Data were collected with a sampling rate of 30Hz and the actigraph was placed on the wrist of the arm with the greatest mobility. If both arms were equally mobile, it was placed on the wrist of the dominant hand. If the legs were more mobile it was placed on the ankle of the most mobile leg. Actigraphs recorded continuously and were only taken off if the patient was bathed, which however only happened once during the “study week” if at all. A critical point in the acquisition of actigraphy data in this patient population is that part of the data will not reflect movements initiated by the patient, but nursing activities and therapies, that is external movements. Especially nursing activities follow a regular schedule, i.e. patients are moved every four hours with body care taking place at these times as well and more extensive body care taking place in the mornings and the evenings. Likewise, therapies and mobilization exclusively occur during the day. The regular pattern of these events might be enough to suggest certain rhythmicity or even a circadian rhythm in actigraphy data. Importantly, earlier studies have not taken this into account or corrected for external movements, probably due to technical limitations (Cruse et al., 2013). We therefore recorded all events in the patient’s room during the “study week” using an app (<https://github.com/wolli2710/HospitalTracker>) that had been developed specifically for this purpose. This allowed us to clean the data removing artefacts resulting from external movements. Using the app, clinical and research staff as well as visitors could indicate the type of activity that was performed by simply tapping the screen of a tablet in the patient’s room (e.g. “nursing”, “patient in wheelchair”, “therapy”, “visit”, etc.). Upon tapping the screen a time stamp was generated, which eventually allowed for a correction of the data.

Cleaning and analysis of actigraphy data was done using R version 3.4.2 (R Core Team, 2015). Following the integration of actigraphy and tablet data into one single dataset, the actigraphy data were downsampled to 1/60 Hz, i.e. one value per minute. The actigraphy values of the time stretches during which clinical staff or visitors were with the patient, the patient was put into a wheelchair or back into bed, the times during which the CRS-R assessments took place as well as the times when the actigraphs had been taken off for body care were removed. The first half of the removed values was replaced by the median activity during the 10min preceding the event (i.e. nursing, therapy, etc.). Likewise, the second half was replaced by the median activity during the 10min following the end of the event. Importantly, we had to impute missing data because calculation of the interdaily stability (IS, see below) of the actigraphy data requires a dataset without missing data. Importantly, to correct for the clinical staff or visitors having indicated their presence too late, we removed and imputed an additional 5min before and after each nursing activity and an additional 10min before and after the start of each visit or when the patient was seated in a wheelchair. This automatic artefact correction was followed by a visual screening and the correction of residual artefacts. This way, we obtained datasets that were assumed to be free from external movements with the remaining activity representing “true” motor activity of the patient.

The resulting datasets were then used to calculate the interdaily stability (IS) of the activity. IS is a non-parametric measure that reflects how well the patients’ activity rhythms were entrained to a 24h zeitgeber (i.e. the light-dark cycle) as indexed by values ranging between 0 for Gaussian noise and 1 for perfect IS. The calculation of this measure is implemented in the R package ‘nparACT’ (for methodological details see Blume, Santhi, & Schabus, 2016).

***Physiological Assessments and Data Analysis***

*Skin Temperature*

Skin temperature was sampled at a rate of 1/300 Hz, i.e. one sample every five minutes, using external skin sensors (iButton DS1922L; Maxim Integrated Products, Inc., San Jose, CA, USA). These temperature sensors have been found to be useful for circadian rhythm research and to give rise to valid results in earlier studies (Hasselberg, McMahon, & Parker, 2013; van Marken Lichtenbelt et al., 2006). A total of four sensors were placed on patients’ skin, two in a proximal location in the infraclavicular fossa about 7-10 cm left and right of the sternum. Additionally, two sensors were placed in a distal position just above the ankles at the insides of the lower legs (cf. Kräuchi, Cajochen, Werth, & Wirz-Justice, 1999; Kräuchi, Cajochen, Werth, & Wirz-Justice, 2000). Sensors were affixed using adhesive medical tape and the skin was shaved beforehand if considered necessary to prevent a painful removal of the tape. One additional sensor was fixed to a bedside table in the patient’s room to record ambient room temperature.

For data processing and analysis we used R version 3.4.3 (R Core Team, 2015). The pre-processing and analysis approach of the temperature data was the same as in Blume et al. (2017). In a first step, data from each sensor were processed individually and artefacts, which resulted from the sensors being taken off for bathing, were removed. Specifically, all values that were outside 2.5 times the interquartile difference from the temperature median of a sensor were removed from the data.

Following data cleaning, we pooled the data from the two proximal and the two distal sensors and computed the proximal-distal skin temperature gradient (DPG) by subtracting the distal from the proximal values in a pointwise fashion. This gradient has been shown to parallel changes in core body temperature and can therefore serve as a proxy for it in patient populations where core body temperature cannot be measured rectally due to medical and potential ethical problems (e.g. Hasselberg et al., 2013; Kräuchi et al., 1999). More precisely, core body temperature is regulated by two mechanisms that complement each other, the regulation of core body temperature itself and heat loss in the periphery (Aschoff & Wever, 1958; Kräuchi, 2002). The interplay between these two contributing factors is mirrored by variations in the DPG, wherefore it serves as a proxy for core body temperature. Following the computation of the DPG we subtracted the grand average from the gradient in a pointwise manner to account for the “offset” before the application of periodogram analyses (see next paragraph) as has been recommended by Van Dongen, Olofsen, Van Hartevelt, and Kruyt (1999).

In a next step, we calculated Lomb-Scargle periodograms (Lomb, 1976; Scargle, 1982) which the DPG served as the input for. This is a least squares spectral analysis (LSSA) method that can be used to detect the rhythmicity underlying time series data such as fluctuations in body temperature. In the process, sine waves of different period lengths are fitted to the data. In the calculation of Lomb-Scargle periodograms the “normalised power” informs about the fit of a sine wave with a specific period length. The “normalised power” is maximal where the sum of squares of the fit of a sinusoid to the data is minimal. The method has been shown to be especially useful for the detection of rhythms in noisy data and allows for an easy computation of levels of significance for each period length. Another advantage is that the Lomb-Scargle periodogram can handle unequally sampled data, i.e. data that contains missing values. This is especially important when the dataset requires artefact-rejection (for a comprehensive discussion of the advantages see Ruf, 1999). We calculated the period length of each patient’s body temperature rhythm using the “lomb” package available for R (Ruf, 1999). We looked for significant peaks in the normalised power of the periodogram between 24 ± 12 hours (i.e. lower limit at 12 hours, upper limit at 36 hours) to ensure we would even detect rhythmicity that substantially deviates from 24 hours. We chose the oversampling factor to be 100 for a fine-grained scanning of frequencies, the significance level was set to α = 0.01. Usually, this approach yielded several significant peaks suggesting that sinusoids with different period lengths substantially contributed to the data. Previous studies have found the unmasked endogenous period of the human temperature rhythm to be 24.18 hours on average (Czeisler et al., 1999), wherefore we selected the peak closest to 24.18 hours (i.e. the “circadian peak”) and declared the corresponding period length the period length of the patient’s circadian temperature rhythm.

For the scheduling of the multiple CRS-R assessments according to the temperature maximum, we also calculated the time of occurrence of this maximum. To this end, in a first step we computed the mean of each sampling point across 24h (i.e. all available values obtained at 3:00pm, at 3:05pm, at 3:10pm etc. were averaged yielding a total of 144 data points) from the DPG data. Subsequently, we averaged data points across consecutive 30min periods (i.e. from 3:00pm to 3:30pm, from 3:05pm to 3:35pm, etc.) to obtain a more reliable estimate of low-frequency changes in body temperature. Finally, we looked for the maximal temperature among all 30min averages and noted the time of occurrence. We then scheduled up to five CRS-R assessments on each of two consecutive days (cf. section “Behavioural Assessment” and Fig. 1).

*Urinary 6-sulfatoxymelatonin (aMT6s)*

For the analysis of circadian variations in melatonin we analysed variations in 6-sulfatoxymelatonin (aMT6s), the major metabolite of melatonin measureable in urine, whose levels are delayed by 2 hours relative to melatonin (Middleton, 2013). Melatoninsulfate is the major metabolite of melatonin in urine. The specific advantage is that, if patients have a catheter, it can be sampled continuously across day and night without interfering with patients’ circadian rhythms for example by waking them up. To this end, we took urine samples every two hours starting at 9am on day 5 of the “study week” until 7am on day 7, i.e. during 48 hours. This ideally resulted in a total of 24 samples; in two patients we however were only able to obtain 23 and 16 samples, respectively, because the catheter was blocked. All patients had a transurethral indwelling catheter ensuring a constant flux of urine and each sample was taken from the urine collected during the preceding two hours. Although estimating the circadian period length from aMT6s is less accurate than from saliva or plasma, it is ideal for circadian research because the samples can be taken at very low light levels without having to wake patients up or even touching them. When samples had been taken, they were stored at room temperature until the last sample had been taken and then frozen at -20°C. For analysis, samples were shipped to Guildford, UK for further analysis.

Analysis of aMT6s levels was done by Stockgrand Ltd. (Guildford, UK) using a radioimmunoassay. The limit of detection for the assay was 0.18 ng/ml, with interassay variation coefficients of (mean ± SD) of 5.7% at 3.3 ± 0.19 ng/ml, 7.8% at 15.5 ± 1.21 ng/ml, and 6.1% at 28.3 ± 1.72 ng/ml.

In a next step, we fit a baseline cosine function (BCF, see Suppl. Formula 1) to the data. While classically, single or complex cosine curves are often fitted to melatonin data, it may occur that they do not yield adequate model curves or lead to wrong estimates of cycle duration and amplitude. This is mainly due to variations in melatonin generally not following a cosine wave, because hormone levels are close to a stable but low baseline during the day with the pineal gland secreting melatonin only during the night. The BCF, originally described by Ruf (1996), in contrast, has been designed to fit rhythmical deviations from a stable baseline in time series data and is essentially “cutting off” part of a cosine wave at a certain (baseline) level.

$$aMT6s\left( t \right)=b+ \frac{H}{2 \times(1-c)}\times(\cos\left( t-\varphi\right)-c+\left| \cos\left( t-\varphi\right)-c \right|)$$

**Suppl. Formula 1:** The Baseline Cosine Function, where 6-sulfatoxymelatonin (aMT6s) levels vary as a function of time (t), φ indicates the acrophase, b the baseline level and H is the height of the sinusoidal peak (in the case of melatonin above) the baseline.

Later studies using human data revealed that the BCF provides a much better and parsimonious description of melatonin profiles than classical approaches. Moreover, they seem to be more robust to noise as well as missing data (for a comprehensive discussion see van Someren & Nagtegaal, 2007). Fitting of the BCF as well as subsequent computations of the dim light melatonin onset (DLMO) was done in R version 3.4.2. For curve fitting as well as the calculation of the goodness of fit statistics we followed the example in Ruf (1996, pp. 164-165) and used the R function ‘constrOptim’. While the initial guess for c was set to 0.2, initial guesses for the parameters φ, b and H we flexibly adapted for each optimisation/ dataset as the optimisation would otherwise sometimes not converge. The initial guess for φ was the time when aMT6s levels were maximal, the guess for b was the minimum of aMT6s levels and for the peak height H we used the sum of baseline and median aMT6s levels. Following the fit of the curve, the DLMO was defined as the time point where aMT6s levels are equal to 25% of the peak height H, which represents a common definition of the DLMO (cf. e.g. Santhi et al., 2016). From the BCF fit, the parameter that was included in model 1 (CRS-R & circadian indexes) was R^2^, i.e. the goodness of fit index that informs about the percentage of variance in the data that could be explained by the fitted BCF. The fitted curves for all participants can be seen in Fig. 2B. For a detailed overview of the BCF fit results please see Suppl. Table. 2. Please note that although the result of the BCF fit was not good enough to reach significance in one and was only marginally significant in another participant, we did not exclude these patients from further analyses as the small R^2^ reflects the poor fit.

***Statistical Analyses***

For all analyses we used R version 3.4.3 (R Core Team, 2015) For statistical analyses of multilevel models we used the “lme4” package.(Bates, Mächler, Bolker, & Walker, 2015) Prior to model fitting discrete variables were z-transformed to allow for an interpretation of the results in terms of how a change of one *SD* on a predictor variable will affect the criterion. For fixed effects we report *t*-values along with degrees of freedom (*df*) rounded to the next integer as the Satterthwaite approximation results in decimals. We also report 95% confidence interval for the fixed effects, which were computed using the “lme4” bootstrapping approach. Effects with *p*-values < .05 are denoted significant and effects with .05 < *p* < .1 are denoted trends. In the following we will briefly describe the three models that were calculated. For a detailed description please see the supplementary material.

***Model Descriptions***

*Model 1: Patient’s State & Circadian Indexes of Body Temperature, Actigraphy and Melatonin*

We assessed the relationship between the behavioural state of the patient (CRS-R sum score) and indexes of circadian rhythm integrity of (i) body temperature, (ii) actigraphy and (iii) melatonin using multilevel modelling. The aim was to keep the model as parsimonious as possible while including all variables deemed relevant. Specifically, we modelled the behavioural state, i.e. the CRS-R sum score deemed most representative during the “study week”. The diagnosis (i.e. UWS or [E]MCS) was modelled as a random intercept because we expected that circadian indexes might differ between diagnosis groups regarding their predictive power. For body temperature, actigraphy and melatonin we included an index of circadian rhythmicity each that was assumed to best predict the outcome as a fixed effect. For skin temperature variations we included the absolute deviation of the period length from 24.18 hours, i.e. the period length found in healthy individuals (Czeisler et al., 1999), which has previously been shown to be related to the state of the patient (cf. Blume et al., 2017). For actigraphy data we included the interdaily stability (IS). This non-parametric index has been suggested to be a sensitive indicator of the entrainment of the rest-activity rhythm to the light-dark cycle (van Someren et al., 1999). The circadian melatonin/ 6-sulfatoxymelatonin rhythm was modelled by the Fisher-z-transformed R^2^ of the baseline cosine function (BCF) fit to the data. R^2^ reflects the stability of the melatonin secretion across two days thus representing an additional index of circadian rhythm integrity. Besides these effects, we also included the patients’ age at the time of the injury as well as the aetiology (i.e. TBI vs. NTBI) as fixed effects. The aetiology of an injury has previously been shown to be related to the prognosis and diagnosis of a patient with traumatic injuries often being related to a better outcome (e.g. Bagnato et al., 2016). Also the age at the time of injury has been suggested to be related to outcome and thus diagnosis (Luauté et al., 2010).

*Models 2a-c: Variation of Patients’ Behavioural State with Time Offset from the Temperature Maximum and Time of Day*

In a second model we assessed whether the patients’ behavioural state varied as a function of time offset from the temperature maximum as well as the time of day. To this end, we modelled the results (i.e. sum scores) of repeated CRS-R assessments at up to five time points during the day. The offset from the temperature maximum has previously been suggested to be related to the patients’ state (Blume et al., 2017) as has time of day (Cortese et al., 2015). As in *Model 1* we included aetiology as well as the age at incident as fixed factors in the model while allowing intercepts to vary between patients. Moreover, we included the time offset from the temperature maximum as a linear and quadratic trend (*Model 2a*). As it has previously been suggested that also daytime is related to CRS-R results while daytime and time offset from the temperature maximum are correlated, we calculated the same model but replaced the time offset from the temperature maximum by daytime in *Model 2b* (cf. Cortese et al., 2015). Additionally, we computed a third *Model 2c* (see below), where we included daytime as well as the linear and quadratic trends of the time offset from the temperature maximum. Specifically, in *Model 2c* we sought to evaluate whether daytime and time offset from the temperature maximum, which are naturally correlated, would still have (an independent) predictive value.

**Suppl. Table 1.** Detailed results of the fit of the baseline cosine function (BCF) to the melatoninsulfate (aMT6s) data.

| **Patient ID** | **Baseline (b) in ng/ml** | **Peak Height (H) in ng/ml** | **R^2^ in %** | ***p*-value** | **DLMO (clocktime)** | **Peak Phase (clocktime)** |
| --- | --- | --- | --- | --- | --- | --- |
| P1 | 2.07 | 11.18 | 81.82 | < .001 | 07:11 | 09:09 |
| P2 | 2.46 | 36.48 | 76.54 | < .001 | 03:12 | 07:18 |
| P3 | 1.23 | 8.00 | 83.80 | < .001 | 02:31 | 06:55 |
| P4 | 1.95 | 3.56 | 48.53 | 0.004 | 02:59 | 06:43 |
| P5 | 2.29 | 7.83 | 81.82 | < .001 | 03:40 | 07:45 |
| P6 | 2.26 | 8.42 | 68.39 | < .001 | 22:09 | 03:40 |
| P7 | 4.29 | 16.61 | 74.73 | < .001 | 03:18 | 05:12 |
| P8 | 2.99 | 34.91 | 93.70 | < .001 | 03:43 | 07:05 |
| P9 | 1.78 | 19.78 | 73.68 | < .001 | 04:55 | 06:57 |
| P10 | 2.03 | 8.46 | 57.50 | < .001 | 01:54 | 05:20 |
| P11 | 0.44 | 1.61 | 19.25 | 0.22 | 21:46 | 04:02 |
| P12 | 1.31 | 36.61 | 87.68 | < .001 | 02:26 | 05:34 |
| P13 | 1.27 | 10.81 | 82.30 | < .001 | 02:51 | 06:01 |
| P14 | 0.60 | 3.24 | 28.40 | 0.08 | 01:23 | 08:54 |
| P15 | 1.51 | 9.36 | 75.24 | < .001 | 19:48 | 00:41 |
| P16 | 2.57 | 28.56 | 77.90 | < .001 | 01:27 | 05:31 |
| P17 | 3.26 | 38.63 | 63.36 | < .001 | 03:16 | 06:06 |
| P18 | 5.55 | 5.21 | 59.66 | < .001 | NA | 09:19 |
| P19 | 10.35 | 81.42 | 78.70 | < .001 | 04:34 | 06:35 |
| P21 | 0.72 | 0.71 | 42.50 | 0.01 | NA | 10:31 |

Abbreviations: R^2^ = Determination coefficient; DLMO = dim light melatonin onset.

**Suppl. Table 2.** Detailed results of the analysis of the actigraphy data.

| **Patient ID** | **Interdaily Stability** | **Intradaily Variability** |
| --- | --- | --- |
| P1 | 0.2 | 1.49 |
| P2 | 0.15 | 0.74 |
| P3 | 0.21 | 1.60 |
| P4 | 0.19 | 1.37 |
| P5 | 0.23 | 1.79 |
| P6 | ̶ | ̶ |
| P7 | 0.14 | 1.29 |
| P8 | 0.15 | 1.35 |
| P9 | 0.21 | 0.67 |
| P10 | 0.18 | 1.91 |
| P11 | 0.37 | 0.88 |
| P12 | 0.13 | 1.22 |
| P13 | 0.37 | 0.97 |
| P14 | 0.23 | 1.49 |
| P15 | 0.17 | 1.90 |
| P16 | 0.15 | 1.82 |
| P17 | 0.20 | 0.49 |
| P18 | ̶ | ̶ |
| P19 | 0.16 | 1.16 |
| P20 | 0.18 | 1.40 |
| P21 | 0.33 | 1.13 |
| P22 | 0.08 | 0.41 |
| P23 | 0.13 | 0.99 |

Abbreviations: Interdaily Stability is a score ranging between 0 and 1 informing about how well the patients’ rest-activity cycles were entrained to the light-dark cycle. Intradaily variability quantifies the fragmentation of the rest-activity pattern. It converges to zero for a perfect sine wave and approaches two for Gaussian noise. However, it may be even higher if an ultradian component with a period length of two hours is present.

**Suppl. Table 3:** Detailed Results of the CRS-R Assessments during the Study Week

| **Patient ID** | **Aetiology** | **Diagnosis** | **CRS-R**  **Σ Score** | **CRS-R**  **Auditory** | **CRS-R**  **Visual** | **CRS-R Motor** | **CRS-R Oromotor/**  **Verbal** | **CRS-R Comm-unication** | **CRS-R Arousal** |
| --- | --- | --- | --- | --- | --- | --- | --- | --- | --- |
| P1 | NTBI | VS/UWS | 6 | 2 | 0 | 2 | 1 | 0 | 1 |
| P2 | TBI | VS/UWS | 5 | 1 | 0 | 2 | 1 | 0 | 1 |
| P3 | NTBI | VS/UWS | 5 | 1 | 0 | 2 | 1 | 0 | 1 |
| P4 | NTBI | VS/UWS | 4 | 0 | 0 | 1 | 1 | 0 | 2 |
| P5 | NTBI | VS/UWS | 6 | 1 | 0 | 2 | 1 | 0 | 2 |
| P6 | TBI | VS/UWS | 2 | 0 | 1 | 0 | 0 | 0 | 1 |
| P7 | NTBI | VS/UWS | 5 | 1 | 0 | 1 | 1 | 0 | 2 |
| P8 | TBI | MCS | 13 | 3 | 5 | 2 | 1 | 0 | 2 |
| P9 | TBI | MCS | 17 | 3 | 5 | 5 | 1 | 1 | 2 |
| P10 | NTBI | VS/UWS | 3 | 0 | 0 | 1 | 1 | 0 | 1 |
| P11 | TBI | VS/UWS | 4 | 0 | 0 | 1 | 1 | 0 | 2 |
| P12 | NTBI | VS/UWS | 4 | 1 | 0 | 1 | 1 | 0 | 1 |
| P13 | NTBI | MCS | 13 | 2 | 3 | 4 | 1 | 0 | 3 |
| P14 | TBI | MCS | 9 | 1 | 3 | 3 | 0 | 0 | 2 |
| P15 | TBI | EMCS | 23 | 4 | 5 | 6 | 3 | 2 | 3 |
| P16 | NTBI | VS/UWS | 5 | 1 | 0 | 1 | 1 | 0 | 2 |
| P17 | TBI | MCS | 9 | 1 | 3 | 2 | 1 | 0 | 2 |
| P18 | TBI | VS/UWS | 2 | 0 | 0 | 0 | 0 | 0 | 2 |
| P19 | NTBI | VS/UWS | 3 | 1 | 0 | 0 | 0 | 0 | 2 |
| P20 | NTBI | MCS | 7 | 1 | 3 | 1 | 1 | 0 | 1 |
| P21 | TBI | VS/UWS | 5 | 0 | 0 | 2 | 2 | 0 | 1 |
| P22 | NTBI | VS/UWS | 2 | 0 | 0 | 1 | 0 | 0 | 1 |
| P23 | TBI | EMCS | 20 | 4 | 5 | 5 | 1 | 2 | 3 |

**Suppl. Table 3** shows the detailed maximal CRS-R scores during the study week. Abbreviations: MCS = Minimally Conscious State; EMCS = Exit MCS; VS/UWS = Vegetative State/ Unresponsive Wakefulness Syndrome; TBI = Traumatic Brain Injury; NTBI = Non-Traumatic Brain Injury.

**Suppl. Table 4:** Detailed Results of the Multiple CRS-R Assessments According to the Temperature Maximum

| **Patient ID** | **TempMax Time** | **Time of Day** | **Diagnosis** | **CRS-R**  **Σ Score** | **CRS-R**  **Auditory** | **CRS-R**  **Visual** | **CRS-R Motor** | **CRS-R Oromotor/**  **Verbal** | **CRS-R Comm-unication** | **CRS-R Arousal** |
| --- | --- | --- | --- | --- | --- | --- | --- | --- | --- | --- |
| P1 | 16:50 | 2/2/3/**4**/5 | U/U/U/**U**/U | 5/5/5/**5**/6 | 1/1/1/**1**/1 | 0/0/0/**0**/0 | 2/2/2/**2**/2 | 1/1/1/**1**/1 | 0/0/0/**0**/0 | 1/1/1/**1**/2 |
| P2 | 16:20 | 2/2/3/**4**/4 | U/U/U/**U**/U | 4/2/6/**4**/3 | 1/0/1/**1**/1 | 0/0/0/**0**/0 | 2/2/2/**0**/0 | 0/0/1/**1**/0 | 0/0/0/**0**/0 | 1/0/2/**2**/2 |
| P3 | 13:40 | 1/**2**/3/4/5 | U/**U**/U/U/U | 6/**5**/5/4/5 | 1/**1**/1/0/1 | 0**/0**/0/0/0 | 2/**2**/2/1/1 | 1/**1**/1/1/1 | 0/**0**/0/0/0 | 2/**1**/1/2/2 |
| P4 | 12:10 | 1/**3**/3/4/5 | U/**U**/U/U/U | 5/**5**/5/6/6 | 1/**1**/1/1/1 | 0/**0**/0/0/0 | 2/**2**/2/2/2 | 0/**1**/1/1/1 | 0**/0**/0/0/0 | 2/**1**/1/2/2 |
| P5 | 14:50 | 2/3/**4**/4/5 | U/U/**U**/U/U | 5/4/**3**/3/2 | 1/0/**0**/0/0 | 0/0/**NA**/0/0 | 2/1/**1**/1/1 | 1/1/**1**/1/1 | 0/0/**0**/0/0 | 1/2/**1**/1/0 |
| P7 | 06:05 | 1/2/3/4/5 | U/U/U/U/U | 3/4/3/2/3 | 0/1/1/0/0 | 0/0/0/0/0 | 0/0/0/0/0 | 1/1/0/0/1 | 0/0/0/0/0 | 2/2/2/2/2 |
| P8 | 21:15 | 2/2/3/4/5 | U/U/M/U/M | 4/4/7/5/9 | 1/1/0/1/2 | 0/0/3/0/3 | 1/1/2/1/1 | 1/1/1/1/1 | 0/0/0/0/0 | 1/1/1/2/2 |
| P9 | 16:00 | 1/2/3/**4**/5 | M/M/M/**M**/M | 8/8/12/**9**/11 | 1/0/3/**0**/3 | 3/3/4/**4**/3 | 2/2/2/**2**/2 | 1/1/1/**1**/1 | 0/0/0/**0**/0 | 1/2/2/**2**/2 |
| P11 | 12:10 | 2/**3**/3/4/5 | U/**U**/U/U/U | 4/**4**/4/3/4 | 0/**0**/0/0/0 | 0/**0**/0/0/0 | 1/**1**/1/1/1 | 1/**1**/1/1/1 | 0/**0**/0/0/0 | 2/**2**/2/1/2 |
| P16 | 16:30 | 2/2/3/**4**/5 | U/U/U/**U**/U | 3/3/4/**4**/4 | 1/1/1/**1**/1 | 0/0/0/**0**/0 | 1/0/1/**1**/1 | 0/0/0/**0**/0 | 0/0/0/**0**/0 | 1/2/2/**2**/2 |
| P17 | 17:50 | 1/2/3/4/**4** | M/M/M/M/**M** | 8/8/8/8/**8** | 1/1/1/1/**1** | 3/3/3/3/**3** | 1/1/1/1/**1** | 1/1/1/1/**1** | 0/0/0/0/**0** | 2/2/2/2/**2** |
| P19 | 13:50 | 1/2/**3**/4/5 | U/U/**U**/U/U | 4/3/**4**/3/4 | 1/1/**1**/0/1 | 0/0/**0**/0/0 | 1/0/**0**/1/1 | 0/0/**1**/1/0 | 0/0/**0**/0/0 | 2/2/**2**/1/2 |
| P20 | 11:55 | 1/**2**/3/4/4 | U/**M**/M/M/M | 3/**9**/7/7/7 | 0/**3**/1/1/1 | 1/**3**/3/3/3 | 1/**1**/1/1/1 | 0/**1**/0/0/1 | 0/**0**/0/0/0 | 1/**1**/2/2/1 |
| P21 | 10:15 | 2/**2**/3/4/5 | U/**M**/U/M/M | 4/**8**/4/6/8 | 0/**4**/1/4/4 | 0/**0**/0/0/0 | 1/**2**/1/1/1 | 1/**1**/1/0/1 | 0/**0**/0/0/0 | 2/**1**/1/1/2 |
| P22 | 18:15 | 1/2/3/4/**5** | U/U/U/U/**U** | 3/2/2/4/**2** | 0/0/0/1/**0** | 0/0/0/0/**0** | 1/0/0/1/**0** | 0/0/0/0/**1** | 0/0/0/0/**0** | 2/2/2/2/**1** |
| P23 | 14:00 | 2/2/**3**/4/5 | E/E/**E**/E/E | 20/20/**20**/20/20 | 4/4/**4**/4/4 | 5/5/**5**/5/5 | 5/5/**5**/5/5 | 1/1/**1**/1/1 | 2/2/**2**/2/2 | 3/3/**3**/3/3 |

**Suppl. Table 4** shows the detailed CRS-R scores when patients were tested multiple times around to the time point of occurrence of the temperature maximum. Patients could be tested at the following time points before and after the temperature maximum -12.5 hrs/ -10 hrs/ -7.5 hrs/ -5 hrs/ -2.5 hrs/ TempMax/ +2.5 hrs/ +5 hrs/ +7.5 hrs/ +10 hrs/ +12.5 hrs. The “Time of Day” gives the daytime at which a patient was tested: 1 = 6-9am; 2 = 9-12am; 3 = 12-3pm; 4 = 3-6pm; 5= 6-9pm. As assessments only took place in these five time intervals, a maximum of five scores were obtained in each patient. The diagnoses and sum scores obtained during assessments at the time of the temperature maximum are marked in bold. The assessment results to the left/right of the rating in bold represent further assessments that were scheduled in intervals of 2.5 hours around the time of the maximum. Please note that for P7 and P8 the temperature maximum occurred at times when an assessment was not possible. Abbreviations: M = Minimally Conscious State; E = Exit MCS; U = Vegetative State/ Unresponsive Wakefulness Syndrome.

**Supplementary Results**

***Group Differences***

We assessed group differences in circadian skin temperature period length, fit of the baseline cosine curve (BCF) to the data and interdaily stability (IS) of actigraphy (dependent variables) between CONSCIOUSNESS states (VS/UWS vs. MCS/ MCS_exit_) as well as the AETIOLOGY subgroups (traumatic brain injury [TBI] and non-traumatic brain injury [NTBI]) using advanced non-parametric approaches as implemented in the “npmv” package (see Burchett, Ellis, Harrar, & Bathke, 2017 for methodological and mathematical details). Here, we report the ANOVA type test with permutation test *p*-values (50000 permutations).

*Skin Temperature: Circadian Period Length*

No group differences between VS/UWS and (E)MCS patients were evident for the period length of the skin temperature cycle (*ATS*_1, 15.5_ = 0.025, *p* = .87, *mdn*_VS/UWS_ = 24.14, *mdn*_(E)MCS_ = 23.96). For an illustration of the distribution of period lengths see suppl. Figure S1.


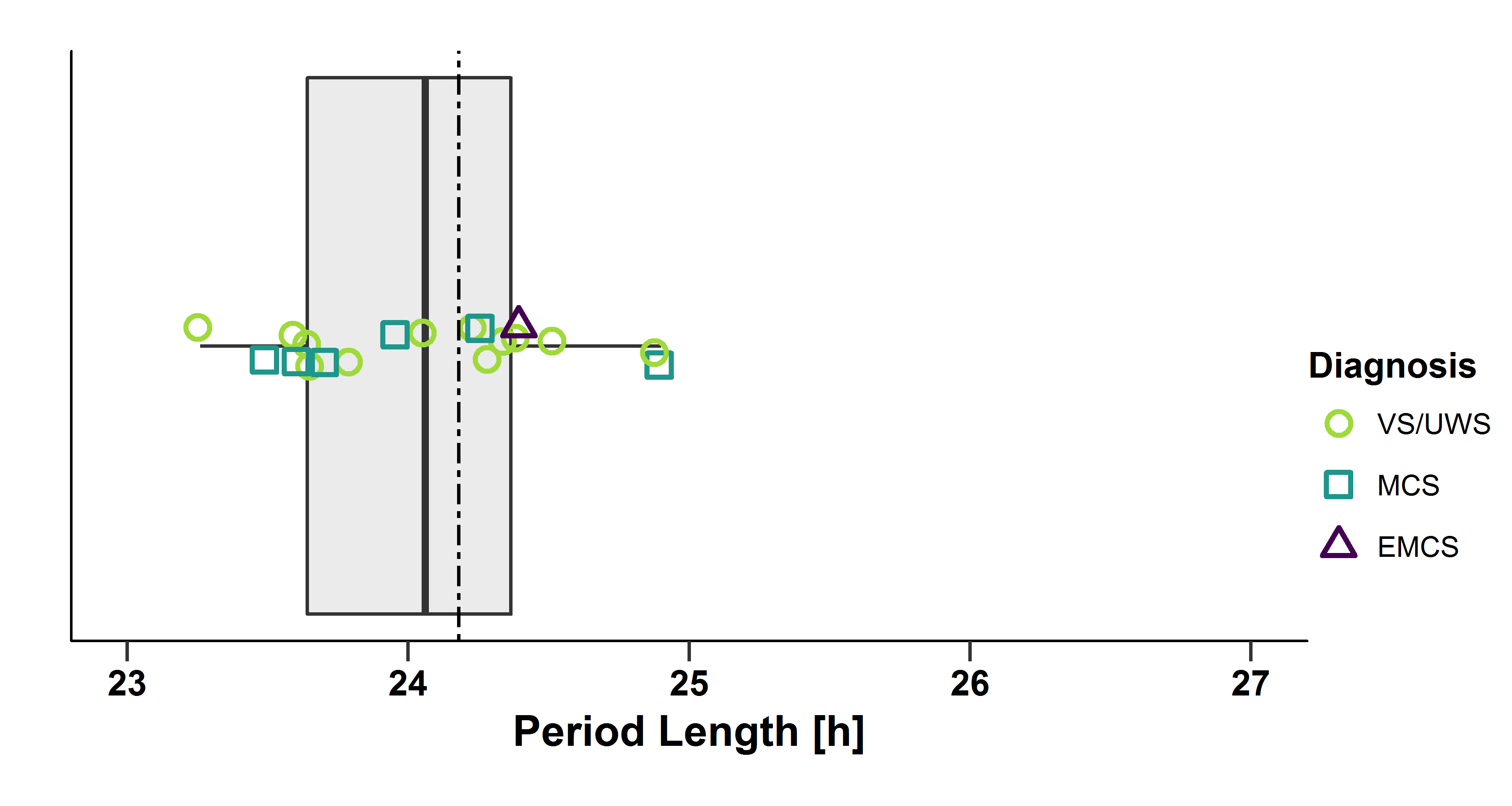


**Fig. S1**: **Boxplot of the distribution of period lengths of circadian temperature rhythms according to the consciousness state.** The dashed vertical line indicates 24.18h, i.e. the “ideal” period length reported in well-controlled studies on healthy individuals. The box shows the quartiles, the vertical line in the box represents the median. Whiskers of the boxplot indicate the 1st and 3rd quartile ± 1.5 times the interquartile difference (IQD).

There were also no differences between the two aetiology groups, i.e. NTBI and TBI patients (*ATS*_1, 16.5_ = 0.63, *p* = .43, *mdn*_NTBI_= 24.22, *mdn*_TBI_ = 23.74). For an illustration of the distribution of period lengths see Figure S2.


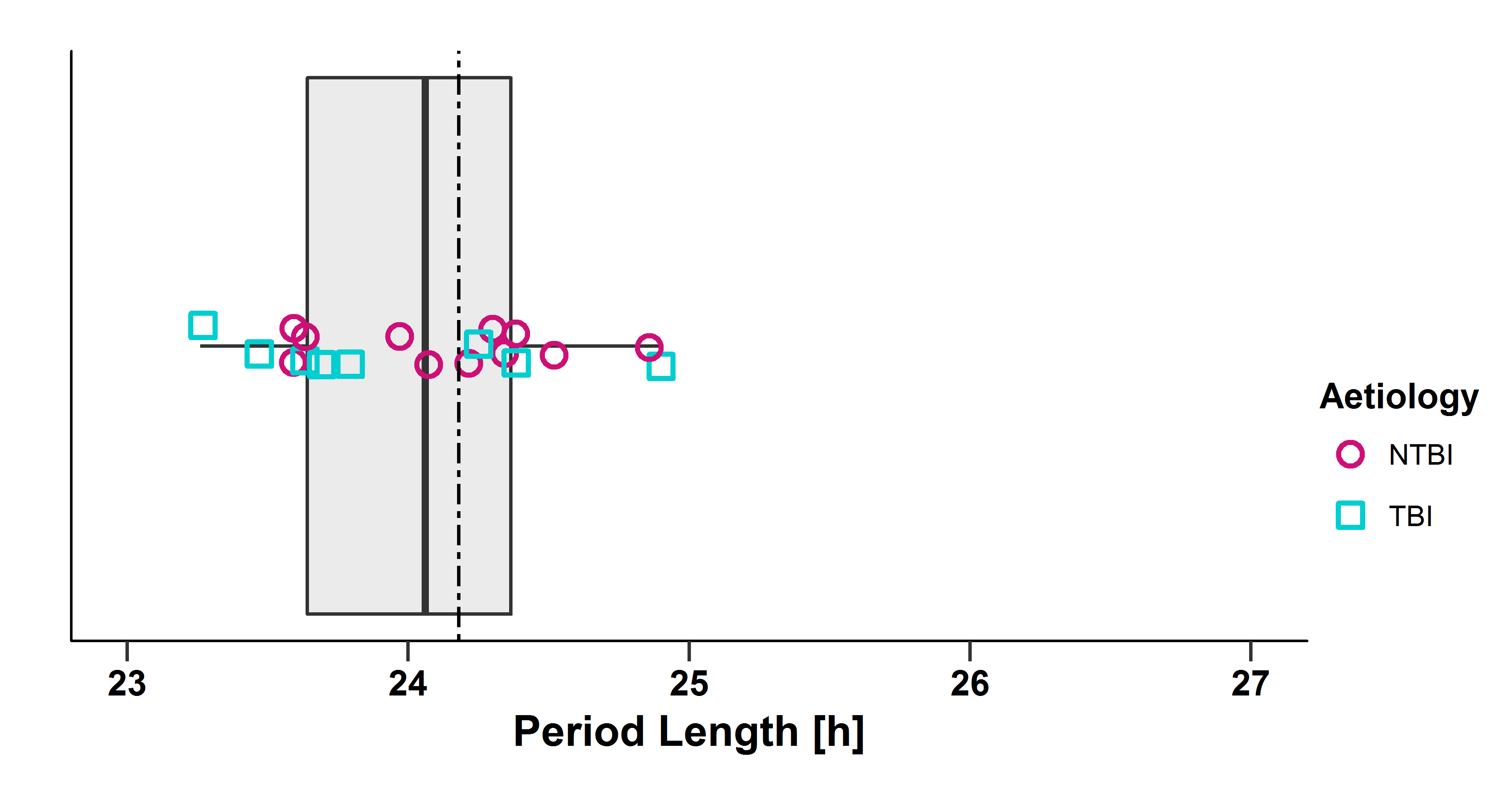


**Fig. S2**: **Boxplot of the distribution of period lengths of circadian temperature rhythms according to the aetiology.** The dashed vertical line indicates 24.18h, i.e. the “ideal” period length reported in well-controlled studies on healthy individuals. The box shows the quartiles, the vertical line in the box represents the median. Whiskers of the boxplot indicate the 1st and 3rd quartile ± 1.5 times the interquartile difference (IQD).

*Melatonin: Fit of the Baseline Cosine Function*

Also for the fit of the baseline cosine function (BCF) to the sulfatoxymelatonin (aMT6s) data we did not find differences between VS/UWS and (E)MCS patients (*ATS*_1, 16_ = 0.11, *p* = .74, *mdn*_VS/UWS_ = 76.54, *mdn*_(E)MCS_ = 73.68, cf. Fig. S3).


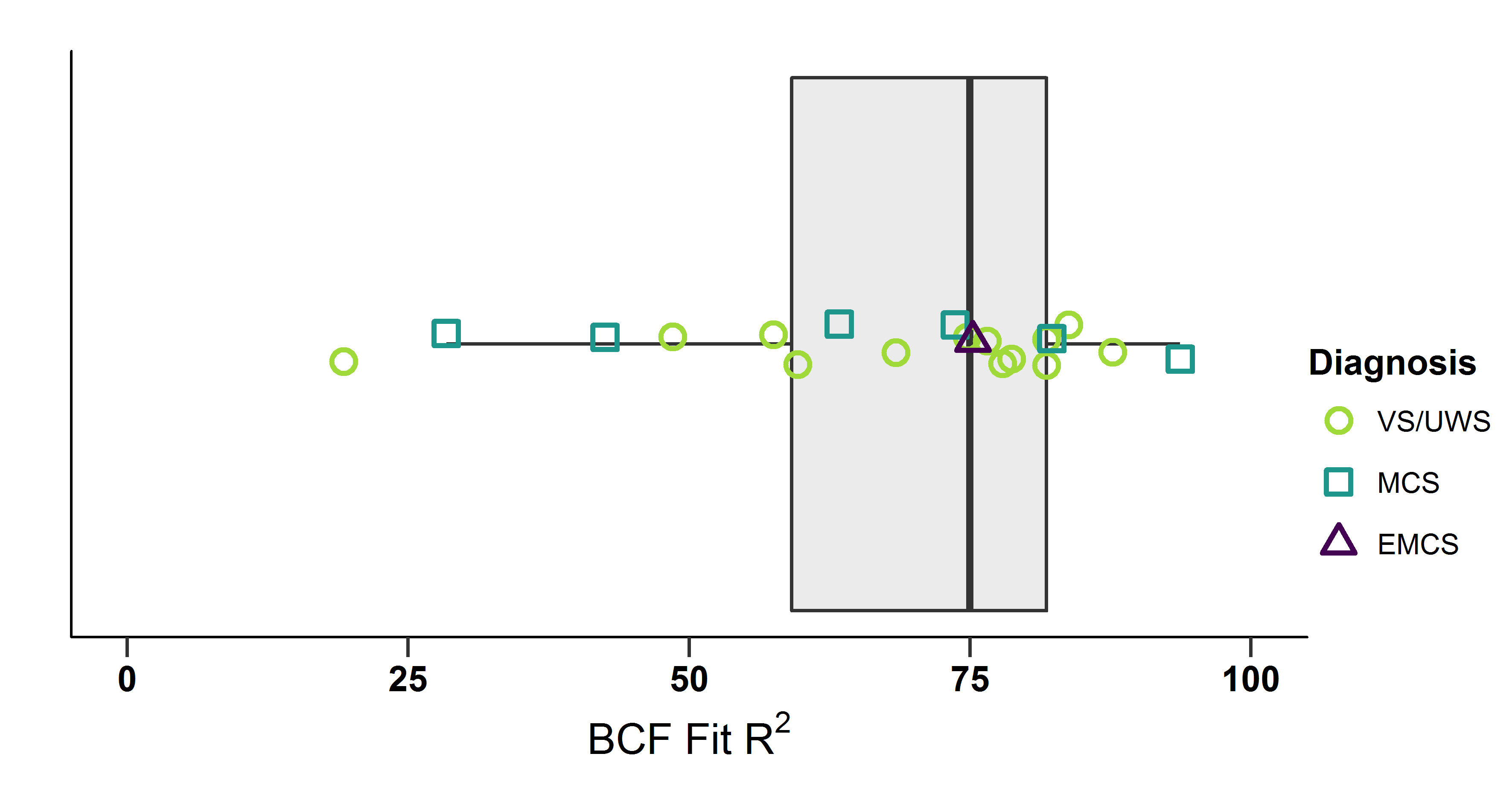


**Fig. S3**: **Boxplot of the distribution of the fit of the baseline cosine function according to the consciousness state.** The x-axis shows the BCF fit in terms of R^2^ (in %). The box shows the quartiles, the vertical line in the box represents the median. Whiskers of the boxplot indicate the 1st and 3rd quartile ± 1.5 times the interquartile difference (IQD).

Neither did we find significant differences between aetiology groups (*ATS*_1, 17.8_ = 2.1, *p* = .17, *mdn*_NTBI_= 78.70, *mdn*_TBI_ = 68.39, cf. Suppl. Fig. S4).


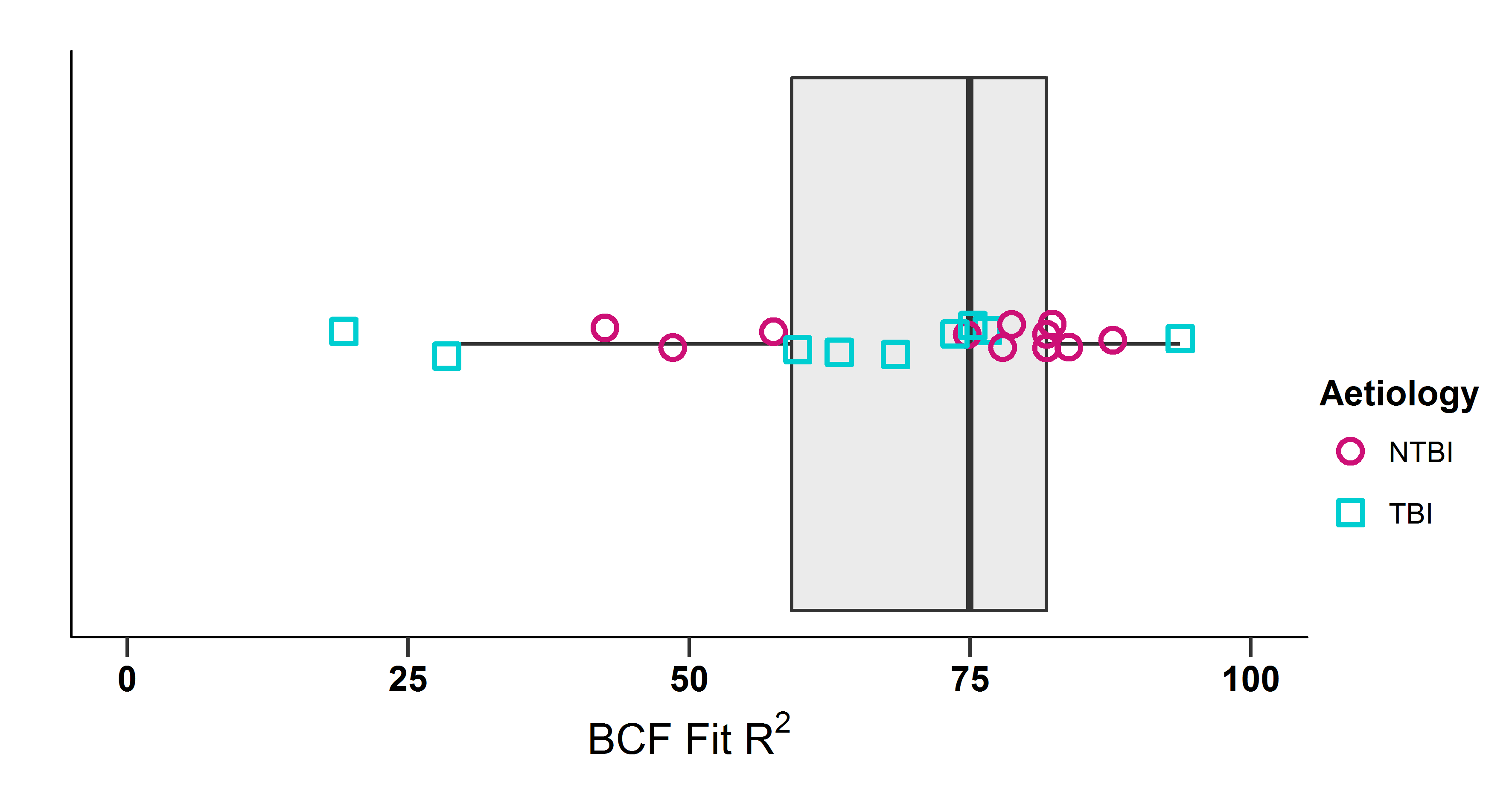


**Suppl. Fig. S4**: **Boxplot of the distribution of the fit of the baseline cosine function according to the aetiology.** The x-axis shows the BCF fit in terms of R^2^ (in %). The box shows the quartiles, the vertical line in the box represents the median. Whiskers of the boxplot indicate the 1st and 3rd quartile ± 1.5 times the interquartile difference (IQD).

*Actigraphy: Interdaily Stability (IS)*

No differences were evident between VS/UWS and (E)MCS patients regarding the interdaily stability (IS) of actigraphy patterns (*ATS*_1, 15_ = 1.07, *p* = .31, *mdn*_VS/UWS_ = 0.18, *mdn*_(E)MCS_ = 0.2, cf. Suppl. Fig. S5).


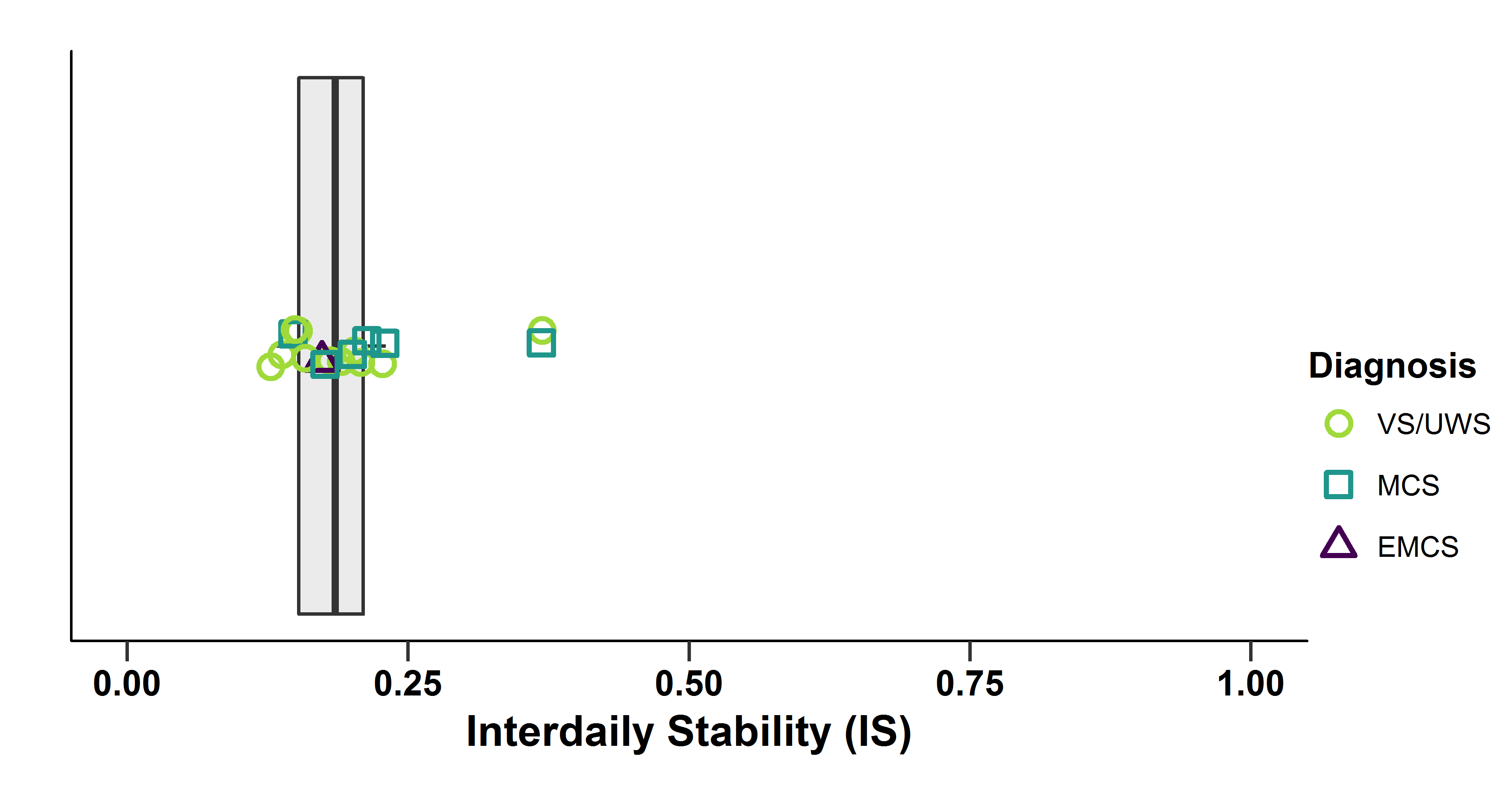


**Fig. S5**: **Boxplot of the distribution of the interdaily stability (IS) of actigraphy patterns according to the diagnosis.** IS informs about how well a patient’s activity rhythm is entrained to a 24h zeitgeber, i.e. the light-dark cycle (range 0-1, 0 indicates Gaussian noise whereas 1 indicates perfect entrainment). The box shows the quartiles, the vertical line in the box represents the median. Whiskers of the boxplot indicate the 1st and 3rd quartile ± 1.5 times the interquartile difference (IQD).

There was also no difference in IS between aetiology groups, i.e. NTBI and TBI patients (*ATS*_1, 15_ = .33, *p* = .56, *mdn*_NTBI_ = 0.18, *mdn*_TBI_ = 0.2, cf. Suppl. Fig. S6).


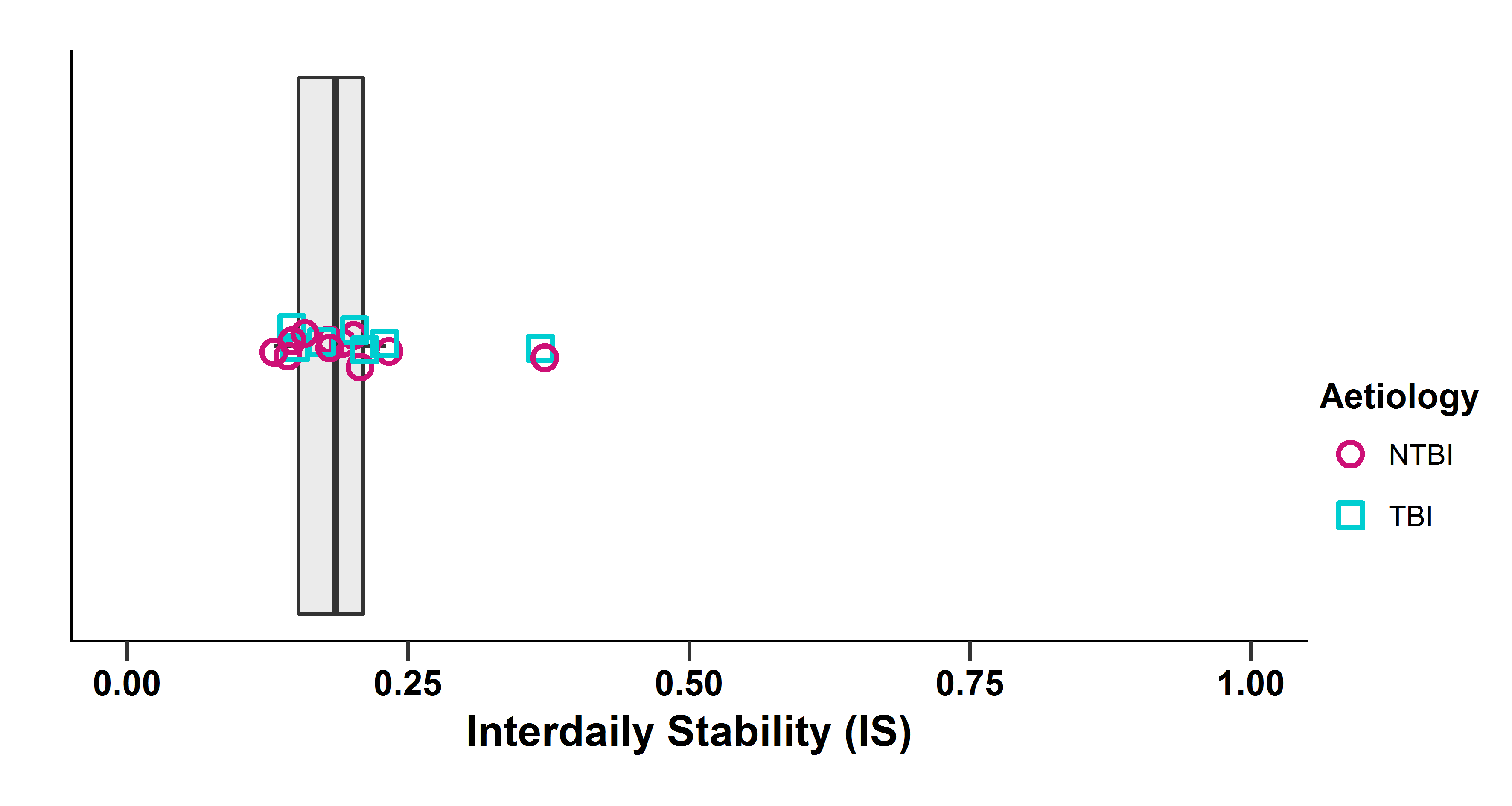


**Fig. S6**: **Boxplot of the distribution of the interdaily stability (IS) of actigraphy patterns according to the aetiology.** IS informs about how well a patient’s activity rhythm is entrained to a 24h zeitgeber, i.e. the light-dark cycle (range 0-1, 0 indicates Gaussian noise whereas 1 indicates perfect entrainment). The box shows the quartiles, the vertical line in the box represents the median. Whiskers of the boxplot indicate the 1st and 3rd quartile ± 1.5 times the interquartile difference (IQD).

Coefficients of determination (R^2^) as well as partial R^2^ statistics for fixed effects are reported in the supplementary material and were calculated using the “r2glmm” package (Nakagawa and Schielzeth method; Jaeger, 2017). Supplementary Table 4 provides an overview of the results as well as the contribution of each factor to *Model 1* in terms of explained variance (*R^2^*).

**Suppl. Table 5**: Effects of the studied predictors on the behavioural state of the patient as measured by the CRS-R explained by a change of group membership (dichotomous variables) or a 1SD increment in the predictor variable (continuous variables). The table also informs about the variance explained by the full model as well as the partial explained variance for each fixed effect (*R^2^*) (Model 1).

| **Effect** | **b** | | **SE (b)** | | **95% CI** | | **R^2^ (95% CI)** | |
| --- | --- | --- | --- | --- | --- | --- | --- | --- |
| Aetiology (NTBI vs. TBI) *** | 6.09 | | 1.46 | | 3.03, 9.20 | | 0.33 (0.05-0.66) | |
| Age at Incident (years) ** | 2.35 | | 0.77 | | 0.84, 3.88 | | 0.17 (0.002-0.53) | |
| Temperature: Deviation of Period Length from 24.18 hrs + | -3.11 | | 1.67 | | -6.35, 0.15 | | 0.07 (0-0.41) | |
| Actigraphy (IS) | 0.64 | | 0.59 | | -0.51, 1.79 | | 0.02 (0-0.33) | |
| Melatonin: BCF Fit *** | 2.80 | | 0.64 | | 1.54, 4.09 | | 0.29 (0.26-0.62) | |
| Full Model |  | |  | |  | | 0.42 (0.22-0.75) | |
|  |  |  | |  | |  | |  |

Abbreviations: NTBI = non-traumatic brain injury; TBI = traumatic brain injury; IS = interdaily stability; BCF = baseline cosine function; b = standardised regression coefficient; SE = Standard Error; CI = Confidence Interval; *R^2^* = (partial) determination coefficient; +*p* < .01, **p* <.05, ***p* <.01, ****p* <.001.

Supplementary Tables 6-8 provide an overview of the effects of the studied predictors on the behavioural state of the patient as measured by the CRS-R explained by a change of group membership (dichotomous variables) or a 1 *SD* increment in the predictor variable (continuous variables). The tables also informs about the variance explained by the full model as well as the partial explained variance for each fixed effect (*R^2^*) (*Models 2a-c*).

**Suppl. Table 6**: CRS-R scores as predicted by aetiology (NTBI vs. TBI), age at incident and time of day (Model 2a).

| **Effect** | **b** | **SE (b)** | **95% CI** | | **R^2^ (95% CI)** | |
| --- | --- | --- | --- | --- | --- | --- |
| Aetiology (NTBI vs. TBI) ** | 6.39 | 1.97 | 2.55, 10.36 | | 0.44 (0.28-0.60) | |
| Age at Incident (years) * | 2.17 | 1.01 | 0.18, 4.18 | | 0.26 (0.10-0.44) | |
| Daytime (hours) + | 0.23 | 1.13 | -0.03, 0.49 | | 0.01 (0.00-0.11) | |
| Full Model |  |  |  | | 0.45 (0.30-0.61) | |
|  |  |  |  |  | |  |

**Suppl. Table 7**: CRS-R scores as predicted by aetiology (NTBI vs. TBI), age at incident and offset from the temperature maximum as a quadratic function (Model 2b).

| **Effect** | **b** | **SE (b)** | **95% CI** | | **R^2^ (95% CI)** | |
| --- | --- | --- | --- | --- | --- | --- |
| Aetiology (NTBI vs. TBI) ** | 6.36 | 1.98 | 2.40, 10.24 | | 0.44 (0.27-0.60) | |
| Age at Incident (years) + | 2.17 | 1.01 | 0.03, 4.02 | | 0.22 (0.07-0.40) | |
| Offset from Tempmax (hours) + | 0.48 | 0.26 | -0.03, 0.98 | | 0.01 (0.0-0.12) | |
| Offset from Tempmax^2^ + | -0.41 | 0.23 | -0.85, 0.03 | | 0.01 (0.0-0.11) | |
| Full Model |  |  |  | | 0.45 (0.30-0.62) | |
|  |  |  |  |  | |  |

**Suppl. Table 8**: CRS-R scores as predicted by aetiology (NTBI vs. TBI), age at incident, time of day and offset from the temperature maximum as a quadratic function (Model 2c).

| **Effect** | **b** | **SE (b)** | **95% CI** | | **R^2^ (95% CI)** | |
| --- | --- | --- | --- | --- | --- | --- |
| Aetiology (NTBI vs. TBI) ** | 6.36 | 1.98 | 2.53, 10.16 | | 0.44 (0.27-0.60) | |
| Age at Incident (years) + | 2.08 | 1.03 | 0.07, 4.07 | | 0.22 (0.07-0.40) | |
| Daytime (hours) | 0.12 | 0.33 | -0.54, 0.78 | | 0.00 (0.0-0.08) | |
| Offset from Tempmax (hours) | 0.26 | 0.64 | -1.01, 1.52 | | 0.00 (0.0-0.09) | |
| Offset from Tempmax^2^ | -0.34 | 0.29 | -0.90, 0.22 | | 0.01 (0.0-0.10) | |
| Full Model |  |  |  | | 0.45 (0.21-0.62) | |
|  |  |  |  |  | |  |

Abbreviations: NTBI = non-traumatic brain injury; TBI = traumatic brain injury; *b* = standardised regression coefficient; SE = Standard Error; CI = Confidence Interval; *R^2^* = (partial) determination coefficient; +*p* < .01, **p* <.05, ***p* <.01.

**Data Availability**

Anonymised data is available from the corresponding author upon and will be shared upon request from any qualified investigator.

**Acknowledgements**

We thank Sarah Haberl and Julius Köppen as well as the staff at the Albert-Schweitzer-Klinik Graz and the Gunther-Ladurner Pflegezentrum Salzburg for their constant support and help with the data collection. We also thank Dr. Thomas Scherndl for sharing his expertise on multilevel modelling.

**Declaration of Interests**

The authors declare no conflicts of interest.

**Authors’ contributions**

Study design: MaS, CB; Data acquisition: CB, MA, MR, RdG, GP, MoS, ABK; Data analysis and interpretation: CB, MaS, NS; Drafting the manuscript: CB, MaS; Critical revision of the manuscript: MA, MR, NS, RdG, GP, MoS, ABK, ET, MaS.

**Funding**

CB is supported by the Konrad-Adenauer-Stiftung e.V.. CB and MA are funded by a grant from the Austrian Science Fund FWF (Y-777). CB and RdG are additionally supported by the Doctoral College ‘‘Imaging the Mind’’ (FWF; W1233-G17).

**References**

Aschoff, J., & Wever, R. (1958). Kern und Schale im Wärmehaushalt des Menschen. *Die Naturwissenschaften, 45*.

Bagnato, S., Boccagni, C., Sant'Angelo, A., Fingelkurts, A. A., Fingelkurts, A. A., & Galardi, G. (2016). Longitudinal Assessment of Clinical Signs of Recovery in Patients with Unresponsive Wakefulness Syndrome after Traumatic or Nontraumatic Brain Injury. *J Neurotrauma, 34*(2), 535-539. doi:10.1089/neu.2016.4418

Bates, D., Mächler, M., Bolker, B., & Walker, S. (2015). Fitting Linear Mixed-Effects Models Using lme4. *2015, 67*(1), 48. doi:10.18637/jss.v067.i01

Blume, C., Lechinger, J., Santhi, N., Giudice, R. d., Gnjezda, M.-T., Pichler, G., . . . Schabus, M. (2017). Significance of circadian rhythms in severely brain-injured patients: A clue to consciousness? *Neurology*. doi:10.1212/wnl.0000000000003942

Blume, C., Santhi, N., & Schabus, M. (2016). ‘nparACT’package for R: A free software tool for the non-parametric analysis of actigraphy data. *MethodsX*.

Burchett, W. W., Ellis, A. R., Harrar, S. W., & Bathke, A. C. (2017). Nonparametric Inference for Multivariate Data: The R Package npmv. *Journal of Statistical Software, 76*(4), 18. doi:10.18637/jss.v076.i04

Cohen, D. A., Wang, W., Wyatt, J. K., Kronauer, R. E., Dijk, D.-J., Czeisler, C. A., & Klerman, E. B. (2010). Uncovering residual effects of chronic sleep loss on human performance. *Science Translational Medicine, 2*(14).

Cortese, M., Riganello, F., Arcuri, F., Pugliese, M., Lucca, L., Dolce, G., & Sannita, W. (2015). Coma recovery scale-r: variability in the disorder of consciousness. *BMC Neurol, 15*(1), 186.

Cruse, D., Thibaut, A., Demertzi, A., Nantes, J. C., Bruno, M.-A., Gosseries, O., . . . Laureys, S. (2013). Actigraphy assessments of circadian sleep-wake cycles in the Vegetative and Minimally Conscious States. *BMC Med, 11*(1), 18.

Czeisler, C. A., Duffy, J. F., Shanahan, T. L., Brown, E. N., Mitchell, J. F., Rimmer, D. W., . . . Emens, J. S. (1999). Stability, precision, and near-24-hour period of the human circadian pacemaker. *Science, 284*(5423), 2177-2181.

Hasselberg, M. J., McMahon, J., & Parker, K. (2013). The validity, reliability, and utility of the iButton (R) for measurement of body temperature circadian rhythms in sleep/wake research. *Sleep medicine, 14*(1), 5-11. doi:DOI 10.1016/j.sleep.2010.12.011

Jaeger, B. (2017). r2glmm: Computes R Squared for Mixed (Multilevel) Models (Version 0.1.2). Retrieved from <https://CRAN.R-project.org/package=r2glmm>

Kalmar, K., & Giacino, J. T. (2005). The JFK coma recovery scale - revised. *Neuropsychological Rehabilitation, 15*(3-4), 454-460. doi:10.1080/09602010443000425

Kräuchi, K. (2002). How is the circadian rhythm of core body temperature regulated? *Clinical Autonomic Research, 12*(3), 147-149.

Kräuchi, K., Cajochen, C., Werth, E., & Wirz-Justice, A. (1999). Physiology - Warm feet promote the rapid onset of sleep. *Nature, 401*(6748), 36-37. doi:Doi 10.1038/43366

Kräuchi, K., Cajochen, C., Werth, E., & Wirz-Justice, A. (2000). Functional link between distal vasodilation and sleep-onset latency? *American Journal of Physiology - Regulatory, Integrative and Comparative Physiology, 278*(3), R741-R748.

Lomb, N. R. (1976). Least-squares frequency analysis of unequally spaced data. *Astrophysics and space science, 39*(2), 447-462.

Luauté, J., Maucort-Boulch, D., Tell, L., Quelard, F., Sarraf, T., Iwaz, J., . . . Fischer, C. (2010). Long-term outcomes of chronic minimally conscious and vegetative states. *Neurology, 75*(3), 246-252. doi:10.1212/WNL.0b013e3181e8e8df

Middleton, B. (2013). Measurement of Melatonin and 6-Sulphatoxymelatonin. In M. J. Wheeler (Ed.), *Hormone Assays in Biological Fluids* (pp. 171-199). Totowa, NJ: Humana Press.

R Core Team. (2015). R: A Language and Environment for Statistical Computing. Vienna, Austria: R Foundation for Statistical Computing. Retrieved from <https://www.R-project.org>

Ruf, T. (1996). The baseline cosinus function: a periodic regression model for biological rhythms. *Biological Rhythm Research, 27*(2), 153-165.

Ruf, T. (1999). The Lomb-Scargle periodogram in biological rhythm research: analysis of incomplete and unequally spaced time-series. *Biological Rhythm Research, 30*(2), 178-201.

Santhi, N., Lazar, A. S., McCabe, P. J., Lo, J. C., Groeger, J. A., & Dijk, D.-J. (2016). Sex differences in the circadian regulation of sleep and waking cognition in humans. *Proc Natl Acad Sci U S A, 113*(19), E2730-E2739. doi:10.1073/pnas.1521637113

Scargle, J. D. (1982). Studies in astronomical time series analysis. II-Statistical aspects of spectral analysis of unevenly spaced data. *The Astrophysical Journal, 263*, 835-853.

Van Dongen, H. P., Olofsen, E., Van Hartevelt, J. H., & Kruyt, E. W. (1999). Searching for biological rhythms: peak detection in the periodogram of unequally spaced data. *Journal of biological rhythms, 14*(6), 617-620.

van Marken Lichtenbelt, W. D., Daanen, H. A., Wouters, L., Fronczek, R., Raymann, R. J., Severens, N. M., & Van Someren, E. J. (2006). Evaluation of wireless determination of skin temperature using iButtons. *Physiology & Behavior, 88*(4), 489-497.

van Someren, E. J. W., & Nagtegaal, E. (2007). Improving melatonin circadian phase estimates. *Sleep medicine, 8*(6), 590-601.

van Someren, E. J. W., Swaab, D. F., Colenda, C. C., Cohen, W., McCall, W. V., & Rosenquist, P. B. (1999). Bright light therapy: improved sensitivity to its effects on rest-activity rhythms in Alzheimer patients by application of nonparametric methods. *Chronobiology international, 16*(4), 505-518.
